# School-related physical activity interventions and mental health among children: a systematic review and meta-analysis

**DOI:** 10.1186/s40798-020-00254-x

**Published:** 2020-06-16

**Authors:** Susanne Andermo, Mats Hallgren, Thi-Thuy-Dung Nguyen, Sofie Jonsson, Solveig Petersen, Marita Friberg, Anja Romqvist, Brendon Stubbs, Liselotte Schäfer Elinder

**Affiliations:** 1 Community Nutrition and Physical Activity, Department of Global Public Health, Karolinska Institutet, Solnavaegen 1E, 104 65 Stockholm, Sweden; 2 Epidemiology of Psychiatric Conditions, Substance Use and Social Environment, Department of Global Public Health, Karolinska Institutet, Solnavaegen 1E, 10465 Stockholm, Sweden; 3 Center for Epidemiology and Community Medicine, Region Stockholm, Solnavaegen 1E, 104 65 Stockholm, Sweden; 4 Unit for Intervention and Implementation Research, Department of Environmental Medicine, Karolinska Institutet, Nobels vaeg 13, 17165 Solna, Sweden; 5 Department of Living Conditions and Lifestyle, The Public Health Agency of Sweden, Nobels väg 18, 171 82 Solna, Sweden; 6 Physiotherapy Department, South London and Maudsley NHS Foundation Trust, Denmark Hill, London, UK; 7 Health Service and Population Research Department, Institute of Psychiatry, Psychology and Neuroscience, King’s College London, De Crespigny Park, London, UK

**Keywords:** Physical activity, Children, Mental health, School-related, Systematic review, Meta-analysis

## Abstract

**Background:**

Low levels of physical activity, sedentary behaviour and mental health problems are issues that have received considerable attention in the last decade. The aim of this systematic review and meta-analysis was to investigate effects of interventions targeting school-related physical activity or sedentary behaviour on mental health in children and adolescents and to identify the features of effective interventions.

**Methods:**

Scientific articles published between January 2009 and October 2019 fulfilling the following criteria were included: general populations of children and adolescents between age 4 and 19, all types of school-related efforts to promote physical activity or reduce sedentary behaviour. Study selection, data extraction and quality assessment were done by at least two authors independently of each other. Data were analysed with a random effects meta-analysis and by narrative moderator analyses.

**Results:**

The literature search resulted in 10265 unique articles. Thirty-one articles, describing 30 interventions, were finally included. Eleven relevant outcomes were identified: health-related quality of life, well-being, self-esteem and self-worth, resilience, positive effect, positive mental health, anxiety, depression, emotional problems, negative effect and internalising mental health problems. There was a significant beneficial effect of school-related physical activity interventions on resilience (Hedges’ *g* = 0.748, 95% CI = 0.326; 1.170, *p* = 0.001), positive mental health (Hedges’ *g* = 0.405, 95% CI = 0.208; 0.603, *p* = < 0.001), well-being (Hedges’ *g* = 0.877, 95% CI = 0.356; 1.398, *p* = < 0.001) and anxiety (Hedges’ *g* = 0.347, 95% CI = 0.072; 0.623, *p* = 0.013). Heterogeneity was moderate to high (*I*^2^ = 59–98%) between studies for all outcomes except positive effect, where heterogeneity was low (*I*^2^ = 2%). The narrative moderator analyses of outcomes based on 10 or more studies showed that age of the children moderated the effect of the intervention on internalising mental health problems. Interventions in younger children showed a significantly negative or no effect on internalising mental health problems while those in older children showed a significant positive or no effect. Moreover, studies with a high implementation reach showed a significant negative or no effect while those with a low level of implementation showed no or a positive effect. No signs of effect moderation were found for self-esteem, well-being or positive mental health. Risk of publication bias was evident for several outcomes, but adjustment did not change the results.

**Conclusions:**

School-related physical activity interventions may reduce anxiety, increase resilience, improve well-being and increase positive mental health in children and adolescents. Considering the positive effects of physical activity on health in general, these findings may reinforce school-based initiatives to increase physical activity. However, the studies show considerable heterogeneity. The results should therefore be interpreted with caution. Future studies should report on implementation factors and more clearly describe the activities of the control group and whether the activity is added to or replacing ordinary physical education lessons in order to aid interpretation of results.

**Trial registration:**

PROSPERO, CRD42018086757.

## Key points

- School-based physical activity interventions may have small beneficial effects on anxiety, resilience, well-being and positive mental health, while the effect of reduced sedentary behaviour on mental health is unclear.
- Future studies should more clearly report on implementation, describe the activities of the control group and whether the activity is added to or replacing ordinary physical education lessons in order to facilitate the interpretation of results.
- It is unclear what type of interventions provides the best effect on mental health and by which mechanisms they work.

## Introduction

Mental health problems have increased among children and adolescents over a number of years in high-income countries, especially in Northern Europe [1], but the reasons for this remain elusive. According to figures from the 2017 Global Burden of Disease study, anxiety and depressive disorders are among the top four leading causes of the disease burden among young people in Western Europe and top six in Sweden [2]. In general, a larger proportion of girls and young women report mental health problems as compared to boys and young men but they all follow the same patterns of increase over time [3]. The National Board of Health and Welfare in Sweden reported that the number of children and adolescents who have received healthcare for depression or anxiety has increased during the period 2006–2016 [4]. An analysis of factors associated with this apparently increasing trend of mental health problems did not specifically point out changes in family or socioeconomic factors, but instead highlighted the issue of increasing stress in school and worries related to further education and career opportunities in the longer perspective as possible factors behind this development [5]. This raises the question of whether schools can intervene to prevent or delay the onset of mental ill-health and/or promote the development of positive mental health defined as a state of well-being where individuals can cope with the normal stresses of life and successfully participate in everyday life [6]. Schools are an effective setting to reach children at no extra cost to the participants and their families. Several school-based psychological universal prevention programmes have been carried out with modest but significantly positive effects on depression in younger children [7] and on depression and anxiety in older children [8].

One type of intervention which has received attention in recent years is physical activity. Physical activity is defined as any bodily movement that gives rise to increased energy expenditure above resting level [9]. Few children and youth reach recommended levels of physical activity worldwide and specifically in high-income countries [9–11], including Sweden [3]. Physical activity can differ according to type of activity e.g. yoga or football, frequency (times per day or week), duration (minutes or hours) and intensity measured by age-related maximum heart rate. Previous reviews have demonstrated beneficial psychological benefits of physical activity such as reductions in levels of depression among children and adolescents [12–14] in addition to their general health promoting effects. Moreover, strong and consistent relationships have been found between sedentary time using screens for leisure and depressive symptomatology and psychological distress, respectively [15]. Prevention programmes can be universal reaching all children or targeted at groups with elevated risk or with clinical symptoms [16]. Targeted interventions usually result in larger effect sizes [17]. Systematic reviews of universal or targeted interventions not restricted to the school setting have concluded that physical activity has beneficial effects on psychosocial outcomes such as externalising [17] and internalising mental health problems [17], self-concept [17, 18], self-esteem [19], academic achievement [17] and overall mental health [14]. Liu et al. [18] reviewed the effects of physical activity interventions mainly involving children with obesity, disability or very inactive children in the school setting. These authors concluded that physical activity had a positive effect on self-concept and self-worth and that the effect was stronger in school-based settings compared to other settings.

To the best of our knowledge, no systematic review has yet been conducted focusing on school-related interventions increasing physical activity or decreasing sedentary behaviour with the aim of improving mental health or reducing mental ill-health in general populations of school children. Therefore, there is a need to systematise current knowledge regarding the effectiveness of school-based physical activity interventions on mental health, to specify the optimal type of interventions and to clarify mechanisms of action. Such knowledge can be used by policy-makers and schools as a basis for actions to promote positive mental health and prevent mental ill-health in school-aged children. The aims of the systematic review were as follows:

1. To study the impact of school-related physical activity interventions or interventions to reduce sedentary behaviour on symptoms of mental health in terms of internalising mental health problems and positive mental health in children aged 4–19 years
2. To investigate possible moderators of these effects such as age, sex, socioeconomic status, family structure, geographical location, focus of the intervention, type of control group, level of implementation and study quality

## Methods

### Study registration and protocol

This review adheres to the Preferred Reporting Items for Systematic Reviews and Meta-Analyses (PRISMA) statement for reporting systematic reviews and meta-analyses [20]. It was registered with the International Prospective Register of Systematic Reviews (PROSPERO; registration no. CRD42018086757) available from: https://www.crd.york.ac.uk/prospero/.

### Search strategy

A literature search was conducted on March 16, 2018, with an updated search on October 24, 2019, using the following databases: MEDLINE; Epub Ahead of Print, In-Process & Other Non-Indexed Citations; Ovid MEDLINE(R); Daily and Ovid MEDLINE (R) (Ovid); PsycINFO (Ovid); Web of Science Core Collection; ERIC (ProQuest); and Sociological Abstracts (ProQuest). Search terms were used to describe the population (e.g. school student), intervention (e.g. physical activity), outcomes (e.g. mental health) and study design (e.g. RCT). Studies were limited to English and Swedish language (see Online resource 1 for the full search strategy). Reference lists from studies meeting inclusion criteria as well as recent reviews in the field were hand-searched.

### Inclusion and exclusion criteria

The criteria for inclusion in the review were peer-reviewed original empirical studies published between January 2009 and October 2019. Studies were included if the population consisted of general population samples of children in preschool, primary school and secondary school, aged 4 to 19 years.

All types of school-related or school-initiated interventions were included. This could be single- or multicomponent interventions, conducted in- or outside school, with a component aiming to increase physical activity or decrease sedentary behaviour. Examples were active breaks during the school day, policies, regulations or environmental changes that can promote physical activity or reduce sedentary behaviour. We included only randomised controlled trials (RCT), cluster-RCTs (cRCT), quasi-experimental or longitudinal observational study designs with a control or comparison group. The comparison group had to come from the same base population or should be matched on key factors and could be a non-exposed group, a physical education-(PE)-as-usual group, a waitlist control group or other-intervention-without-physical-activity group.

Studies were included if they reported any one of the following primary or secondary outcomes at both baseline and post-intervention: Positive mental health defined by well-being, health-related quality of life, happiness, self-esteem, self-confidence, self-compassion, self-efficacy, resilience, positive effect and coping, internalising mental health problems defined by emotional problems, worries, anxiety, negative effect and depressive symptoms. The outcome should be measured by a valid and reliable rating scale suitable for children and adolescents. When more than one relevant outcome was described in the same study, the overall concepts were given priority over subdomains of the concept. Only studies using established and validated measures of the indicated outcomes suitable for children and adolescents were included.

Studies were excluded if they targeted purely clinical populations, if the intervention was not school-related, or the aim was not to increase physical activity or reduce sedentary behaviour. For pragmatic reasons, studies were also excluded if they solely addressed the following aspects of positive mental health and internalising mental health problems (outcomes): self-realisation, working ability, the ability to contribute to society, self-destructive behaviour, problematic eating behaviour and psychosomatic disorders such as recurring pain, sleep problems or stress. Interventions not requiring additional energy expenditure such as mindfulness were also excluded.

### Data extraction

Two authors (S.A. and S.J.) independently screened the titles and abstracts of the identified articles. Articles judged as potentially eligible by at least one author were imported into EndNote Reference Manager, version X6 (Thomson Reuters, Philadelphia, PA) and retrieved for full-text review. Both authors independently read the full text of these articles using the established inclusion and exclusion criteria. Disagreements were resolved through discussion with a third author (L.S.E.). From the included studies, two authors (S.A. and S.J.) independently extracted relevant information into a spreadsheet in Excel with the help of a standardised checklist. Extracted items included main author, year of publication, study design, population characteristics and sample size, characteristics of the intervention, type of control group, relevant mental health outcomes (mean scores and standard deviation (SD) or difference in mean scores and standard error (SE) at baseline and at end of intervention), instruments used, time of measurement and main findings. The extracted data were compared and in case of disagreement, a third author (L.S.E.) checked the data. If relevant data were not included in the article, the corresponding author was contacted and asked to supply the data. If no answer was received after 1 month, a reminder was sent. If no answer was received after additional 2 weeks or if authors were unable to provide the requested data, the paper was excluded and the reason documented. Finally, data were transferred into the Comprehensive Meta-Analysis Software (CMA version 3.0, Biostat. Inc., Englewood NJ, USA) for the meta-analysis. A *p* value of < 0.05 was used to indicate statistical significance.

To capture the implementation of the intervention, the following data were extracted: implementation fidelity, dose, quality, responsiveness, reach and adaption. However, if the information provided in the included articles was not sufficient, the literature was searched for additional publications containing this information. Despite these efforts, the only aspect with enough data to allow for comparisons across studies was reach, i.e. the proportion of children reached by the intervention. Implementation reach was categorised on a scale from 1 to 4 with 1, 80–100% (high); 2, 60–79% (moderate); 3, < 60% (low); and 4, unknown.

### Data preparation

Before the meta-analysis could be conducted, a number of decisions were made regarding which scales and instruments to combine in each outcome and the appropriate method to achieve this. If a study reported results of comparisons for multiple intervention groups with one control group, the combined mean and SD of the intervention groups was calculated before calculating the effect sizes [21]. Likewise, if results were reported separately for boys and girls, we calculated a combined mean and SD. If two relevant scales were used simultaneously in a study population to capture different aspects of the same outcome, a merged mean and SD was calculated for the two outcomes, given the scales had the same metrics [21]. Otherwise, one of the scales was chosen in order to avoid multiple dependent effect sizes within studies, which would assign more weight to studies with more outcomes. The selection of relevant outcomes from each study was done by a consensus procedure between three of the authors (S.A., M.H. and L.S.E.) based on theoretical grounds.

### Meta-analysis

Owing to the anticipated heterogeneity across studies, we conducted a random effects meta-analysis. From each included study, unadjusted mean scores and SD at baseline and follow-up were entered for the intervention and control groups. For studies that did not report unadjusted mean scores, adjusted mean scores or differences in means and SEs were entered. If a study reported results from multiple follow-ups (e.g. post-intervention, 6-months, 12-months), the first follow-up point (post-treatment) was chosen to compare with the baseline score. None of the studies reported within-group correlation (i.e. pre to post-intervention), but we assumed a within-group correlation of 0.7 [21]. Where studies reported the standard error (SE) or confidence interval (CI) instead of the SD, the SD was calculated [21]. The effect size of each included study was calculated by computing mean difference (posttest-pretest) between the intervention and the control group and divided by the pooled standard deviations.

The pooled standardised mean difference (SMD) was then calculated as the difference in mean scores between the intervention and control groups summed across studies. As the SMD is subject to bias due to small sample size [21], we report the corrected SMD (Hedges’ *g*) together with 95% confidence intervals (CIs) and *p* values. A positive value of Hedges’ *g* indicates a positive effect of the intervention, while a negative value indicates the opposite. Values of Hedges’ *g* 0.2, 0.5 and 0.8 represent a small, medium and large effect size, respectively. The *I*^2^ statistic is the proportion of the observed variance that is due to the true between-study variance; i.e. heterogeneity. Values in the order of 25%, 50% and 75% might be considered as low, moderate and high, respectively [22]. Significance can be inferred by the *p* value for heterogeneity, the *Q* statistic. A significant value for *Q* confirms the hypothesis that the true effect size differs across studies.

### Quality assessment of studies

Two authors (S.A. and L.S.E.) independently assessed study quality using the Effective Public Health Practice Project (EPHPP) Quality Assessment Tool for Quantitative Studies [23]. The EPHPP has a rating scale of 1–3 (1 = strong, 2 = moderate and 3 = weak). Quality was assessed on selection bias, study design, confounders, blinding, data collection methods and withdrawal and dropouts. Selection bias was scored based on population representativeness and percentage agreeing to take part. The EPHPP tool does not mention cluster RCT studies but we decided also to award the score ‘strong’ for this study design. Confounders were scored based on reported differences with regard to relevant confounders between groups at baseline and on the percentage of reported confounders controlled for. Blinding was scored based on whether the participants were blinded to the research question, and the assessors were blinded to the group allocation. Data collection was scored based on the evidence reported for validity and reliability of the measurement tools used. Finally, withdrawal and dropout were scored based on the percentage of participants completing the study. A global rating was then determined based on the ratings of the above constructs. A ‘strong’ global rating was awarded if no weak ratings were present, a ‘moderate’ global rating if there was only one weak rating and a ‘weak’ global rating if there were two or more weak ratings. Intervention integrity (assessed by whether the intervention consistency was measured; what percentage received the intervention; was there potential for contamination) and appropriate analysis in relation to the research question(s) (unit of analysis; unit of allocation; statistical analysis; intention to treat) were also assessed. However, the scoring of these constructs did not contribute to the overall rating score.

### Risk of publication bias

To detect the risk of publication bias across studies, we used funnel plots to examine the asymmetric distribution of studies around their mean effect size in the outcome variables, and Egger’s tests for the association between sample sizes and effect sizes that were included in the meta-analysis for each outcome (i.e. tests for asymmetric funnel plots). To quantify the effect of potential publication bias on meta-analytic summary effects, Duval and Tweedie’s trim and fill method was applied if there was significant risk of publication bias. This procedure estimates the summary effect after adjusting for potential publication bias.

### Moderator analysis

Moderator analysis was done narratively. For the narrative analysis, studies were grouped into three categories for each outcome, those with a statistically significant negative (not desired) effect, those with a null effect and those with a statistically significant positive (desired) effect. These groupings were then compared to different levels of the potential moderator, e.g. focus of the intervention.

## Results

### Study selection

The search resulted in 14,821 hits and after removal of duplicates 10,265 unique titles remained. Duplicates were removed via the EndNote Reference Manager software.

**Fig. 1:**
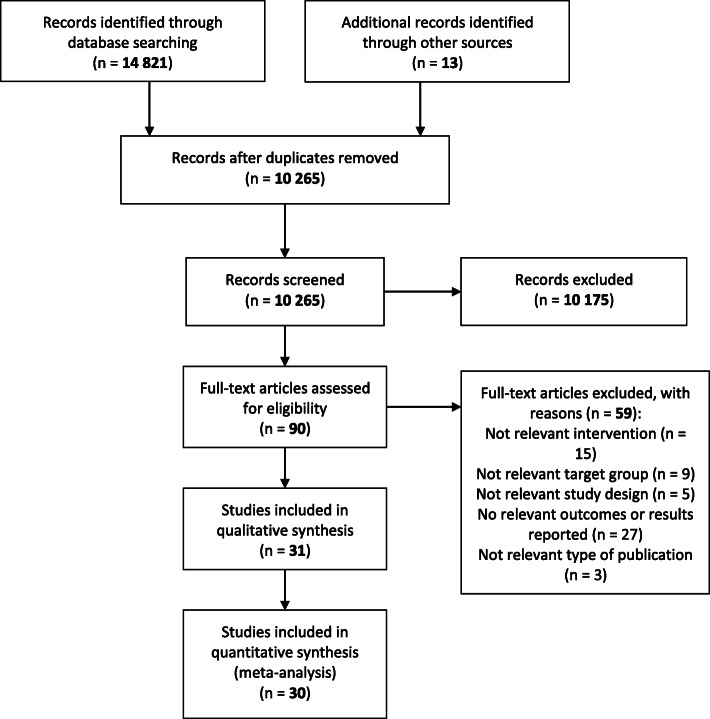
Prisma flow chart

The flowchart is shown in Fig. 1.

Thirty-one articles were included in the analysis, representing 30 different intervention studies [24–54], all of which were published in English. There were three studies by Melnyk et al. and one by Ardic et al. [26, 44–46] representing the same intervention, namely Creating Opportunities for Personal Empowerment (COPE). Two of the studies by Melnyk et al. [44, 46] were from the same intervention, and therefore the long-term follow-up study [46] was not included in the meta-analysis. Studies read in full text and excluded were documented with reasons for exclusion and are shown in Online resource 2.

### Study characteristics

Characteristics of included studies are shown in Table 1. The sample size varied from 19 [45] to 2797 participants. Mean age varied between 8 and 17 years, and the proportion of females varied from 31 to 100%. Socioeconomic status was mixed or low in most studies and unknown in ten studies [25, 28, 33, 35, 39, 40, 43, 50, 53, 54]. Included studies came from twelve countries. Eight studies were conducted in the USA [34, 36, 41, 44–46, 48, 53], and six were from Australia [30, 33, 40, 42, 47, 49]. The rest were conducted in Great Britain (*n* = 5) [24, 27, 32, 37, 38], Ireland (*n* = 2) [29, 52], Germany (*n* = 1) [39], China (*n* = 1) [35], South Korea (*n* = 1) [54], Canada (*n* = 1) [28], Denmark (*n* = 1) [31], Spain (*n* = 2) [43, 51], Norway (*n* = 1) [50] and Turkey (*n* = 2) [25, 26].

**Table 1:** Characteristics of included studies

Study	Study design	Population, sample size, age, sex, SES	Name and description of intervention	Control group	Relevant mental health-related outcomes	Main findings in mental health outcomes as reported	Comment
Adab et al. [24]	cRCT (schools randomised by blocked balanced algorithm)	*n* = 1467 (663 int^a^; 778 cont^b^) students from 26 intervention schools and 28 control schools in the West Midlands region of the UK. Age: 6–7 years (mean age: 6.3) Sex M/F (%) = 51/49 SES: Index of Multiple Deprivation (IMD) median (IQR) score: 38.9 (20.1–49.5)	The WAVES study Content: (1) Additional daily PA during school hours, (2) ‘Villa Vitality’ (interactive healthy lifestyles learning, in an inspirational setting), (3) school-based healthy cooking skills/education workshops for parents and children and (4) information to families with regard to local PA opportunities. Duration: 30 min PA/day for 12 months Deliverer: Research team and school staff	Content: PE as usual with educational resources provided and encouraged to use in schools (but not prescribed). Duration: NR Deliverer: NR	Health-related quality of life; emotional functioning score	No significant between-group effect on health-related quality of life. Subgroup analyses showed no evidence of heterogeneity of treatment effects by sex, ethnicity, household deprivation or baseline weight status.	Data on emotional functioning score obtained from authors
Altunkurek and Bebis [25]	cRCT	*N* = 99 (33 int, 66 cont) from 2 intervention schools and 1 control school in Turkey. Age: 12–15 Sex M/F (%): 47/53 SES: NR	Wellness coaching programme Content: 3-part programme including PA, individual interview and group education Duration: 90 min × 1 session/week over 12 weeks Deliverer: wellness coach researcher	Content: No intervention Duration: NR Deliverer: NR	Wellness	Significant between-group effect (wellness coaching group vs control group) on wellness.	Data from the health education group were not used in this review.
Ardic and Erdogan [26]	Q-exp	*n* = 100 (50 int; 50 cont) students from 1 intervention school and 1 control school in Istanbul, Turkey. Age: 12–15 years (mean age: 12.8) Sex M/F (%) = 50/50 SES: Parents with higher/lower education than secondary school (*n*): 55/119	T-COPE Healthy TEEN programme Content: Healthy lifestyle information, and cognitive behavioural skill building, based on Cognitive behaviour theory (CBT), homework assignments including a journal log capturing participants’ goals and progress, daily use of pedometer Duration: Weekly sessions a 40 min including 10–15 min PA for 15-weeks Deliverer: Research team	Content: Health-related instructions not related to T-COPE, no PA but given instructions on how to use pedometers Duration: 15 weeks Deliverer: NR	Anxiety; depression	Significant between-group effect on anxiety; no significant between-group effect on depression.	
Azevedo et al. [27]	Observational	*n* = 497 (int: 280; cont 217) students from 5 intervention schools and 2 control schools in local urban areas in UK. Age: 11–13 years (mean age: 11.3) Sex M/F (%) = 36/64 SES: Index of Multiple Deprivation (IMD) mean (range) Intervention: 6.8 (1.5–20.3) Control: 17.5 (5.1–30.0); Free school meals eligibility mean (range) Intervention: 40.8 (21.8–52.8) Control: 27.7 (15.4–39.9)	Content: Structured delivery of dance mats into the physical education (PE) classes for six weeks. Thereafter free use of dance mats, though local authority recommended use in scheduled PE classes. Duration: Two hours of physical education lessons per week with use of dance matts. One school provided 1 h and 40 min per week, up to 12 months. Deliverer: School staff	Content: PE as usual Duration: 2 h of physical education lessons per week Deliverer: NR	Psychological well-being	Significant between-group effect on psychological well-being	Data on KIDSCREEN-10 obtained from authors
Bremer et al. [28]	Q-exp	*N* = 362 (265 int, 97 cont) from 19 intervention classes and 11 control class in 7 local Catholic elementary schools in Canada. Age: 9–14 (mean age: 11.7) Sex M/F (%): 51/49 SES: NR	Daily physical activity programme Content: Structure PA including jumping jacks, squats, running, body weight exercises. A 5-km fun run/walk Duration: 20 min /day over 20 weeks Deliverer: teachers	Content: PE as usual Duration: NR Deliverer: NR	Emotional problem, self-esteem, happiness	No significant between-group effect on self-esteem and happiness; no significant between-group effect on emotional problems	Poor adherence, only 4 (21%) reported daily adherence, most engaging in the programme 3–4 days/week
Breslin et al. [29]	cRCT	*n* = 741 (383 int; 357 cont) from 27 schools in Ireland. Age: 8–9 years (mean age: 8.7 years) Sex M/F (%) = 51.5/48.5 SES: Low SES schools identified through Multiple Deprivation Measure	Sport for LIFE: All Island. Content: PA and healthy eating programme, based on Social cognitive theory, including goal setting, problem solving and self-monitoring. Duration: 1 lesson per week for 12 weeks Deliverer: Student volunteers	Content: Waitlist for the programme as well as PE as usual Duration: NR Deliverer: NR	Psychological well-being and HRQOL	No significant between-group effect on psychological well-being	KIDSCREEN total score obtained from authors
Casey et al. [30]	cRCT (schools matched and randomised in pairs paired)	*n* = 614 (358 int; 256 cont) students from 8 intervention schools and 8 control schools in rural and regional communities, Australia. Age: NR (mean age: 13.4) Sex M/F (%) = 0/100 SES: Low SES Australian rural and regional communities	Content: School PE component which incorporated student-centred teaching approaches and behavioural skill development. The PE component involved students participating in two units: sport unit (tennis or football) and recreational unit (YMCA) outside school Duration: Two 6-session units, ranging from 57–100 min each, once a week during 12 months Deliverer: PE teachers and coaches	Content: PE as usual Duration: NR Deliverer: NR	Health-related quality of life; emotional functioning	Significant between-group effect on health-related quality of life after adjustment for baseline scores	Data on emotional functioning score obtained from authors
Christiansen et al. [31]	cRCT (schools randomised)	2797 (1301 int, 1496 cont) from 12 intervention schools and 12 control schools. Age: 10–13 Sex M/F (%): 51/49 SES: Family upper-middle class 41%, middle class 47%, lower-middle class 12%	Physical intervention programme Content: (1) PE classes focusing on skill development, (2) in-class activities (massage and mindfulness), (3) break-time activities (providing bags with equipment to do PA), (4) theme days (involve students in all settings and focus on social climate for PA) Duration: PE class = 4 class × 90 min over 1 year. In-class activities = minimum 2 × 5 min/day over 1 year. Break-time activities = 3 times × 30 min/week over 1 year. Theme day = 3 times over 1 year. Deliverer: teachers	Content: PE as usual Duration: 45 min/day throughout 1 year Deliverer: teachers	Global self-worth	No significant between-group effect on global self-worth	
Corder et al. [32]	cRCT	*n* = 460 (345 int; 115 cont) students from 2 intervention schools and 1 control school in Cambridgeshire, UK. Age: NR (mean age 13.2) Sex M/F (%) = 47/53 SES: Mixed	GoActive Content: Mentors (older adolescents and peer leaders) chose PA activities and students gained points for trying these and got weekly rewards. Teacher had a supportive role and was asked to encourage their class to participate and facilitate students to collect points. Duration: Two weekly sessions during 8 weeks Deliverer: School staff and pupils with support from research team	Content: Waitlist Duration: NR Deliverer: NR	Well-being	Significant between-group effect on well-being	
Costigan et al. [33]	RCT	*n* = 65 (21 AEP (group 1); 22 RAP (group 2); 22 cont) students from 1 secondary school in new South Wales, Australia. Age: 14–16 years (mean age: 15.8) Sex M/F (%) = 69/31 SES: NR	Content: Physical education lessons Group 1 (AEP): HIIT sessions involving gross motor cardiorespiratory exercises (e.g. shuttle runs, jumping jacks and skipping). Group 2 (RAP): HIIT sessions including a combination of cardiorespiratory and body weight resistance training exercises (e.g. shuttle runs, jumping jacks, skipping, combined with body weight squats and push-ups). Duration: 24 sessions, á 8–10 min, three times per week for 8 weeks Deliverer: Research team	Content: PE and lunchtime activities as usual Duration: 8 weeks intervention Deliverer: School staff	Psychological well-being; psychological distress	No significant between-group effects on psychological well-being or psychological distress	
Frank et al. [34]	RCT	*n* = 159 (80 int; 79 cont) students from 1 inner-city school in California, USA. Age: > 13 years (mean age NR) Sex M/F (%) = 53/47 SES: high poverty area	Transformative Life Skills (TLS) Content: Manualized yoga programme with yoga postures, breathing techniques and centring meditation divided in four units focusing on stress management, body and emotional awareness, self-regulation and building healthy relationships Duration: Each unit included 12 lessons delivered in 15-, 30-, or 60-min segments, 3–4 days per week. Intervention lasted one school semester Deliverer: Yoga instructor	Content: ‘Business as usual’ Duration: NR Deliverer: NR	Positive effect; negative effect	No significant between-groups effects on positive or negative effect	
Ha et al. [35]	cRCT (schools matched and randomised in pairs paired)	*n* = 1592 (796 int; 796 cont) students from 10 intervention schools and 10 control schools in Hong Kong, China. Age: NR (mean age: 12) Sex M/F (%) = 46/54 SES: NR	Coca-Cola Rope Skipping STAR Programme Content: Rope skipping programme embedded within school PE curriculum. Package containing skipping materials, ropes, professional skipping training and ambassadors’ support during the research period. Skipping ropes and relevant materials were also available to students during recess and lunch periods. Duration: 4-weeks Deliverer: Research team, PE teachers, student leaders, ambassadors and coaches	Content: Waitlist, PE as usual Duration: 4 weeks Deliverer: Not applicable	Psychological well-being; health-related quality of life	No significant between-group effect on psychological well-being	Data on health-related quality of life (KIDSCREEN-10) obtained from authors
Haden et al. [36]	RCT	*n* = 30 (15 int; 15 cont) students from 1 public school in New York City, USA. Age: 10–11 years (mean age: 10.8) Sex M/F (%) = 57/43 SES: Family income ($) categorised in 9 groups	Content: Ashtanga-informed yoga practice, consisting of physical postures, breathing practices and relaxation techniques. Home practice not prescribed but encouraged, including yoga practice. Duration: 90 min, three times a week for 12 weeks Deliverer: Yoga-teachers	Content: Usual PE classes, including games such as soccer, volley ball and an indoor walking programme Duration: Same frequency and duration as the intervention group Deliverer: PE teachers	Positive effect; negative effect; global self-worth; internalising problems	Significant between-group effect on negative effect to the disadvantage of the intervention (yoga) group. No significant between-group effect on positive effect, global self-worth or internalising problems.	No other significant changes between groups reported by authors. However meta-analysis showed significant negative effects on global self-worth and internalising problems in intervention group compared to control.
Halliwell et al. [37]	RCT	*N* = 344 (190 int, 154 cont) from 4 primary schools in South West England. Age: 9–11 years (mean age: 9.34) Sex M/F (%): 46/54 SES: Had an above average proportion of students with special educational needs and a below average proportion of student eligible for free school meals.	Brief yoga intervention Content: One of 2 usual PE sessions was replaced by a yoga session. Yoga session consisted of simple yoga asanas with focus on breath and relaxation Duration: 1 × 40 min/week over 4 weeks Plus 1 usual PA session Deliverer: Certified female yoga instructor	Content: PE as usual Duration: 2 session/week Deliverer: NR	Positive effect, negative effect	No significant between-group effect on positive and negative effect	
Harrington et al. [38]	cRCT	*n* = 1752 (867 int; 885 cont) from 20 schools in Midlands, UK. Age: 11–14 years (mean age: 12.8 years) Sex M/F (%) = 0/ 100 SES: free school meal eligibility, % (SD): 11.5 (6.1), and index of multiple derivation (IMD) score (SD): 6.7 (2.4). IMD score ranges between 1–10, where 1 is the least deprived and 10 the most deprived.	Girls Active Content: A support framework for schools to change their PA, PE and sport culture including (1) a training day for teachers, including discussions and establishing of peer-leader groups and development of school action plans, (2) Information and marketing material, (3) peer girls’ leadership. Duration: N/R Deliverer: Youth sport trust national tutor and peer leaders in schools	Content: PE as usual Duration: NR Deliverer: NR	Self-esteem and HRQOL	Significant between-group effect on self-esteem at 7 month follow-up; no Significant between-group effect on self-esteem at 14 month follow-up; no Significant between-group effect on HRQOL	Data on HRQOL and self-esteem obtained from authors. We selected 14 month follow-up.
Hyndman et al. [40]	Q-exp	*n* = 275 (123 int; 152 cont) students from 1 intervention school and 1 control school Regional Western Victoria, Australia. Age: 5–12 years (mean age: 7 int; 8.2 cont) Sex M/F (%) = 50/50 SES: NR	Lunchtime Enjoyment Activity and Play (LEAP) Content: Movable/recycled materials for children to use in the school playground. There was no fixed play equipment in the school grounds during the intervention. Five materials were introduced the first week and each week thereafter a maximum of two additional types were introduced. Teacher supervision. Duration: 30 min play at morning break and 30 min at lunchtime for 8 months Deliverer: Not applicable	Content: Access to usual sports equipment and playground equipment and teacher supervision. No access to the movable/recycled materials. Duration: Access to usual equipment during 15 min in the morning break and 45 min lunch break Deliverer: Not applicable	Quality of life (only assessed in children aged 8–12 years)	No between-group effects on quality of life	
Höner and Demetriou [39]	Q-exp	*n* = 516 (297 int; 219 cont) students from 3 intervention schools and 4 control schools in Baden-Württemberg, Germany. Age: NR (mean age: 11.9) Sex M/F (%) = 45/55 SES: NR	Content: Health-promotion PE lessons, mainly consisting of strength and endurance training taught via games and exercises. The lessons combined age-appropriate practical training, theoretical elements and some additional components (e.g. homework and bonus points for various assignments). Duration: 8 lessons lasting 90 min each for 8 weeks Deliverer: PE teachers	Content: Regular PE classes offered by school, including activities such as gymnastics, swimming and traditional ball games Duration: Same as the intervention group Deliverer: PE teachers	Health-related quality of life (total score), emotional well-being (sub-domain), self-worth (sub-domain)	No significant between-group effects on health-related quality of life, emotional well-being or self-worth (self-esteem). No significant differences between boys and girls.	For economic reasons only half of the sample answered the KINDL-R questionnaire
Khalsa et al. [41]	RCT	*n* = 121 (74 int; 47 cont) students from 1 rural secondary school in Massachusetts, USA. Age: 15–19 years (mean age: 16.8) Sex M/F (%) = 58/42 SES: School had a 17 % low-income population	Yoga Ed programme (modified version) Content: Simple yoga postures, breathing exercise, visualisation and games with an emphasis on fun and relaxation. Duration: A typical session included a 5-min initial relaxation, a 5-min warm-up, 15 min of yoga poses and a 5-min closing relaxation. Each session had a theme that was discussed throughout the session by the instructor (e.g. postures, breathing, relaxation, awareness and meditation). Participants attended 2–3 yoga sessions per week for 11 weeks. Sessions were 30–40 min long Deliverer: Yoga instructor	Content: PE as usual Duration: NR Deliverer: NR	Anxiety; depression; self-esteem; test-anxiety; tension/anxiety; depression/dejection; life-satisfaction; resilience	Significant between-group effect on resilience. No significant between-group effects on anxiety, depression or self-esteem. No significant difference between boys and girls	
Lubans et al. [42]	cRCT (schools matched and randomised in pairs)	*n* = 357 (178 int; 179 cont) students from 6 intervention schools and 6 control schools in New South Wales, Australia. Age: 12–14 years (mean age: 13.2) Sex M/F (%) = 0/100 SES: Schools located in low-income communities	NEAT Girls Content: Focus on promoting lifetime physical activities, reducing sedentary behaviours and encouraging low-cost healthy eating. Enhanced school sport sessions, interactive seminars, nutrition workshops, lunchtime physical activity sessions, parental newsletters and text messaging for social support Duration: 76 classes between 30 and 90 min long for 12 months Deliverer: School teachers	Content: Regular PE during the intervention period. Received a condensed version of the intervention at the completion of the study (waitlist). Duration: Same as intervention group Deliverer: PE teachers	Global self-esteem	No significant between-group effect on self-esteem	
Luna et al. [43]	cRcT	*n* = 113 (44 int; 69 cont) students from classes in one school in Spain. Age: 12–15 years (mean age: 13.82) Sex M/F (%) = 57/43 SES: NR	Content: Physical sport education programme based on a sport education model, that included practice of a sport called Ringo. Duration: 2–3 sessions per week for 6 weeks Deliverer: PE teacher	Content: Other physical activity, a PE model developed for the intervention based on traditional collective sports. Duration: 2 sessions per week for 6 weeks Deliverer: PE teacher	HRQOL, positive effect, negative effect, social anxiety	No significant between-group effect on HRQOL, positive effect and social anxiety; significant between-group effect on negative effect	
Melnyk et al. [45]	cRCT	*n* = 19 (12 int; 7 cont) students in 2 classes from 1 urban high school in a metropolitan southwest city, USA. Age: 14–16 years (mean age: 15.5) Sex M/F (%) = 32/68 SES: Mothers education level (*n*): standard college (1); partial college (1); high school graduate (1); partial high school (1); junior high school (8); under 7 years school (5); missing (2). Fathers education level (*n*): high school graduate (2); partial high school (4); junior high school (4); under 7 years school (6); missing (3)	The COPE teen programme Content: The programme consisted of manualized sessions that delivered (a) educational information on leading a healthy lifestyle and (b) cognitive behavioural skills building which included practice and role playing. Content of the educational sessions included (a) creating a healthy lifestyle, (b) strategies to build self-esteem, (c) stress management, (d) goal setting, (e) effective communication, (f) nutrition and (g) physical activity. All children were also given a pedometer to wear every day. Duration: Participants attended 2–3 50 min sessions per week with 15–20 min of physical activity, during 9 weeks with a total of 15 sessions. Deliverer: Research team	Content: Instructions in health topics, not related to COPE TEEN, pedometers were handed out, no PA. Duration: Same frequency and duration as the intervention group Deliverer: NR	Anxiety; depression	No significant between-group effect on anxiety or depression	
Melnyk et al. [44] and Melnyk et al. [46]	cRCT	*n* = 779 (374 int; 433 cont) students from 11 high schools in the southwest region of the USA. Age: 14–16 years (mean age: 14.7) Sex M/F (%): 48/52 SES: Schools were selected for their diversity across, e.g. economic status	The COPE TEEN programme Content: Manualized, educational and cognitive behavioural skills building programme guided by cognitive theory with different content in each COPE session, e.g. self-esteem, stress and coping. Every session also included physical activity, e.g. dancing, walking and kick boxing movements. Daily use of pedometer, homework assignment and parental newsletter Duration: Session lasted for 50 min including 20 min PA once a week for 15 weeks. Deliverer: Health teachers at school	Content: Manualized content not related to COPE TEEN concentrating on common health issues for adolescents, a manual with homework assignments focusing on the topics being covered in class. Parent newsletter sent home to the parents 4 times during the programme. Duration: Same as intervention group Deliverer: Not applicable	Anxiety; depression	No significant between-group differences on anxiety or depression	
Moore et al. [47]	RCT	*N* = 283 (125 int, 158 cont) from 5 secondary schools in NSW Australia. Age: 12–14 years (mean age: 12.76) Sex M/F (%) = 49/51 SES: High 25%, high average 30%, low average 17%, low 28%	Martial arts based intervention Content: Face-to-face group session including: (1) Psycho-education, (2) Warm-up activities (jogging, push-ups, sit-ups), (3) Stretching, (4) Technical martial arts practice and (5) one of the 3 activities pattern practice (choreographed sequence of movements)/ Sparring (tai-chi sticking hand exercise)/ Meditation Duration: 1x 50 min session/week over 10 weeks Deliverer: a registered psychologist and a 2^nd^ Dan/level black-belt taekwondo instructor	Content: Delayed intervention Duration: NR Deliverer: NR	Emotional difficulties, resilience, self-efficacy	Significant between-group effect on resilience and self-efficacy; no significant between-group effect on emotional difficulties	
Noggle et al. [48]	cRCT	*n* = 51 (36 int; 15 cont) students from 3 classes in 1 public high school in rural western Massachusetts, USA. Age: NR (mean age: 17.2) Sex M/F (%) = 41/59 SES: 16.4 % students of the whole school were considered low-income	Content: Kripalu-based yoga programme including 4 key elements of classical yoga: physical exercises and postures, breathing exercises, deep relaxation and meditation techniques. Each session had a theme that was discussed throughout the session by the instructor (e.g. postures, breathing, relaxation, awareness, values and principles). Duration: 30–40 min yoga session, structured to include a 5-min centring, a 5 min warm-up, 15 min of yoga postures/exercises and a 5 min closing relaxation. Participants attended 2–3 yoga sessions a week for 10 weeks (28 yoga session total). Deliverer: Yoga instructors	Content: PE as usual Duration: 30–40 min classes, 2–3 times a week for 10 weeks Deliverer: School PE instructor	Tension-anxiety; depression-dejection; positive effect; negative effect; life purpose and satisfaction; resilience	Significant between-group effect on tension-anxiety (subscale) and negative effect. No significant between-group effect on depression-dejection, resilience or positive effect.	
Olive et al. [49]	cRCT (schools randomised using computer-generated random numbers)	*n* = 821 (445 int; 376 cont) from 13 intervention schools and 16 control schools in the Australian Capital Territory. Age: 7–12 years (mean age: 8.1) Sex M/F (%) = 54/46 SES: Participating schools were in suburbs with SES index higher than the average index of all towns and cities throughout Australia	Specialist-taught Physical education Content: Face-to-face PE lessons, programmed into the school curriculum. Including 5 movement tasks: (1) coordination and agility drills, (2) skill activities, (3) movement challenges and games, (4) dynamic movement control, (5) core movement. Duration: 2 × 50-min sessions/ week over 4 years of elementary school. Deliverer: specialist teachers trained by Bluearth Foundation	Content: PE as usual Duration: 150 min/week PE Deliverer: generalist classroom teacher	Depression	No significant between-group effect on depression	We selected 12-month follow-up for comparability
Resaland et al. [50]	cRCT	*n* = 1229 (620 int/ 582 cont) randomised, 1129 at baseline (596 int; 533 cont) in 57 schools (28 int schools; 29 cont schools) in Norway. Age: 10 years, (mean age: 10.2 years) Sex M/F (%) = Int 52.7/47.3 Cont: 51.4/48.6 SES: NR	Active Smarter Kids (ASK) Content: 165 extra (in addition to usual PE) teacher-led PA per week that included: PA lessons in the playground (90 min/week), PA breaks during academic lessons (25 min/ week) and PA homework (50 min/ week) Duration: 7 months Deliverer: Teachers at school	Content: PE as usual Duration: 135 min/ week Deliverer: NR	Psychological well-being and HRQOL	No significant between-group effect on psychological well-being	Data on HRQOL obtained from authors
Ruiz-Ariza et al. [51]	RCT	*n* = 214 included final sample 184 ( 90 int; 94 cont) students from 4 secondary schools in Andalucia, Spain. Age: 12–14 years (mean age: 13.73) Sex M/F (%) = 53.3/46.7 SES: Mother’s educational level and maternal work	Content: Cooperative high-intensity training (C_HIIT), 4 min warm-up (running, sideways movements and dynamic stretching) 16 min of C-HIIT in four series of exercise, including cardiorespiratory, speed-agility and coordinative training exercises. Duration: 2 × 16 min (20 min including warm-up) sessions per week over 16 weeks. Deliverer: PE teachers	Content: PE as usual with static stretching Duration: NR Deliverer: NR	Well-being	Significant between-group effect on well-being	Data on well-being obtained from authors
Shannon et al. [52]	Q-exp	*n* = 155 (84 int; 71 cont) from 2 schools in Ireland. Age: 8–9 years (mean age: 8.7 years) Sex M/F (%) = 46.5/ 52.9 (data were missing from one child, therefore the total is less than 100) SES: Low SES schools identified through Multiple Deprivation Measure	Healthy Choices Programme based on Self-determination theory. Content: discussions and physical tasks about health benefit of PA, and a ‘Daily Mile’ in addition to usual PE Duration: Weekly hour-long practical sessions, and 15-min walks per day. In total 2 h and 15 min per week for 10 weeks Deliverer: Trained sport student volunteers and classroom teacher	Content: Waitlist for the programme as well as usual PE Duration: NR Deliverer: NR	HRQOL	No significant between-group effect on HRQOL (total score)	Data on Psychological well-being requested from authors, but not obtained. KIDSCREEN total score used for HRQOL
Velez et al. [53]	RCT	*n* = 31 (16 int; 15 cont) students from 1 predominantly Hispanic high school in USA Age: 14–18 years (mean age: 16.14). Sex M/F (%) = 57/43 SES: NR	Content: Supervised guided resistance training programme Duration: At least 30 sessions (3 days/week for 12 weeks), 35–40 min each Deliverer: Researchers	Content: PE as usual and health class Duration: Same as intervention group Deliverer: Researchers	Self-concept (global self-worth)	Significant between-group effect on global self-worth	
Yook [54]	RCT	*n* = 46 (23 int; 23 cont) students from elementary schools in Seoul, Korea. Age: NR (mean age: 11) Sex M/F (%) = 54/46 SES: NA	Content: Combination of yoga, various running activities and kinball (the latter activities named ‘new sport’) Duration: New sport consisted of warm-up (5 min), the main programme (25 min) and cool down (10 min). Both the yoga and new sport activities were separately practised once per week for about 40 min per session. The intervention lasted 8 weeks with running activities the first 4 weeks and Kinball the last 4 weeks. Deliverer: NR	Content: NR Duration: NR Deliverer: NR	Happiness, resilience; self-esteem	No significant between-group differences in happiness, resilience or self-esteem. No significant differences between boys and girls.	Authors did not combine girls and boys. Meta-analysis identified significant differences between intervention and control group on all outcomes when combining girls and boys.

There was a large variation in the content of the interventions from ordinary school physical exercise, sport and recreation, yoga and playground modifications, to more extensive programmes such as COPE. However, no study had the reduction of sedentary behaviour as a primary aim. We categorised the focus of the interventions into four different types as ‘body’ (*N* = 8) [27, 28, 33, 35, 40, 50, 51, 53] ‘body-education’ (*N* = 11) [24, 29–32, 38, 39, 42, 43, 49, 52] ‘body-mind’ (*N* = 6) [34, 36, 37, 41, 48, 54] or ‘body-education-mind’ (*N* = 5) [25, 26, 44–47]. By ‘body’ we mean interventions aimed at improving body strength physical activity. By ‘education’ we refer to interventions containing learning elements, while ‘mind’ means efforts aimed at strengthening mental processes. See Table 2 for a categorisation of other potential effect moderators. The duration of the interventions varied from 4 weeks to 4 years. The level of implementation reach was low in six studies [24, 27, 28, 30, 38, 48], medium in two studies [32, 41], high in eighteen studies [25, 31, 33, 34, 36, 37, 39, 40, 42–47, 49–51, 53] and unknown in five studies [26, 29, 35, 52, 54]. A description of qualitative implementation factors (fidelity, dose delivered or received, responsiveness, level of adaptation) is shown in Online resource 3.

**Table 2:** Potential effect moderators

Study	Intervention focus^**a**^	Implementation reach	Male/female (%)	Age group^**b**^	SES	Study quality	Type of control group
Adab et al. [24]	Body-edu	Low	51/49	Younger	Mixed	Moderate	PE as usual
Altunkurek and Bebis [25]	Body-mind-edu	High	47/53	Older	NR	Weak	PE as usual
Ardic and Erdogan [26]	Body-mind-edu	NR	50/50	Older	Mixed	Moderate	Other activity but not physical
Azevedo et al. [27]	Body	Low	36/64	Younger	Mixed	Weak	PE as usual
Bremer et al. [28]	Body	Low	51/49	Younger	NR	Weak	PE as usual
Breslin et al. [29]	Body-edu	NR	51.5/48.5	Younger	Low	Weak	Waitlist control
Casey et al. [30]	Body-edu	Low	0/100	Older	Low	Moderate	PE as usual
Christiansen et al. [31]	Body-edu	High	51/49	Younger and older	Mixed	Moderate	PE as usual
Corder et al. [32]	Body-edu	Medium	47/53	Older	Mixed	Moderate	Waitlist control
Costigan et al. [33]	Body	High	69/31	Older	NR	Strong	PE as usual
Frank et al. [34]	Body-mind	High	53/47	Older	Low	Moderate	PE as usual
Ha et al. [35]	Body	NR	46/54	Younger	NR	Strong	Waitlist control
Haden et al. [36]	Body-mind	High	57/43	Younger	Mixed	Moderate	PE as usual
Halliwell et al. [37]	Body-mind	High	46/54	Younger	Low	Moderate	PE as usual
Harrington et al. [38]	Body-edu	Low	0/100	Older	Mixed	Moderate	PE as usual
Hyndman et al. [40]	Body	High	50/50	Younger	NR	Weak	PE as usual
Höner and Demetriou [39]	Body-edu	High	45/55	Younger	NR	Moderate	PE as usual
Khalsa et al. [41]	Body-mind	Medium	58/42	Older	Mixed	Moderate	PE as usual
Lubans et al. [42]	Body-edu	High	0/100	Older	Low	Moderate	Waitlist control
Luna et al. [43]	Body-edu	High	57/43	Older	NR	Weak	Other physical activity
Melnyk et al. [45]	Body-mind-edu	High	32/69	Older	Mixed	Weak	Other activity but not physical
Melnyk et al. [44] and Melnyk et al. [46]	Body-mind-edu	High	48/52	Older	Mixed	Moderate	Other activity but not physical
Moore et al. [47]	Body-mind-edu	High	49/51	Older	Mixed	Strong	PE as usual and waitlist control
Noggle et al. [48]	Body-mind	Low	43/57	Older	Mixed	Weak	PE as usual
Olive et al. [49]	Body-edu	High	54/46	Younger	Mixed	Moderate	PE as usual
Resaland et al. [50]	Body	High	52/48	Younger	NR	Strong	PE as usual
Ruiz-Ariza et al. [51]	Body	High	53.3/46.7	Older	Mixed	Moderate	PE as usual
Shannon et al. [52]	Body-edu	NR	46.5/52.9	Younger	Low	Weak	PE as usual and waitlist control
Velez et al. [53]	Body	High	57/43	Older	NR	Weak	PE as usual
Yook et al. [54]	Body-mind	NR	54/46	Younger	NR	Weak	NR

^a^Body refers to interventions aimed at improving strength or fitness, edu refers to interventions containing learning elements, mind refers to interventions aimed at strengthening mental processes

^b^Younger refers to mean age 12 years or younger, and older refers to mean age above 12 years

The control groups received PE as usual (*N* = 21) [24, 25, 27, 28, 30, 31, 33, 34, 36–41, 47–53], attention control programmes without physical activity (*N* = 4) [26, 44–46], other physical activity (*n* = 1) [43] or were a waitlist control (*N* = 4) [29, 32, 35, 42] while for one study [54], the activity of the control group was not reported. The study designs were RCT (*N* = 9) [33, 34, 36, 37, 41, 47, 51, 53, 54], cRCT (*N* = 15) [24, 25, 29–32, 35, 38, 42–46, 48–50], quasi-experimental (*N* = 5) [26, 28, 39, 40, 52] and observational study (*N* = 1) [27].

In total, nine outcomes were identified, based on at least 3 studies each. These were symptoms of depression, anxiety, emotional problems, negative effect, well-being, health-related quality of life, self-esteem and self-worth, positive effect and resilience. In addition, two composite outcomes were defined: internalising mental health problems and positive mental health. Instruments measuring each outcome are presented in Online resource 4 and a definition of these concepts is given in the “Methods” section (inclusion and exclusion criteria).

### Risk of bias within studies

Study quality was weak, moderate or strong (Table 2, details in Online resource 5). Four studies had strong quality [33, 35, 47, 50], 16 had moderate quality [24, 26, 30–32, 34, 36–39, 41, 42, 44, 46, 49, 51] and 11 had low quality [25, 27–29, 40, 43, 45, 48, 52–54]. The main weaknesses were lack of blinding of participants and assessors, and selection bias.

### Meta-analytic results

**Fig. 2:**
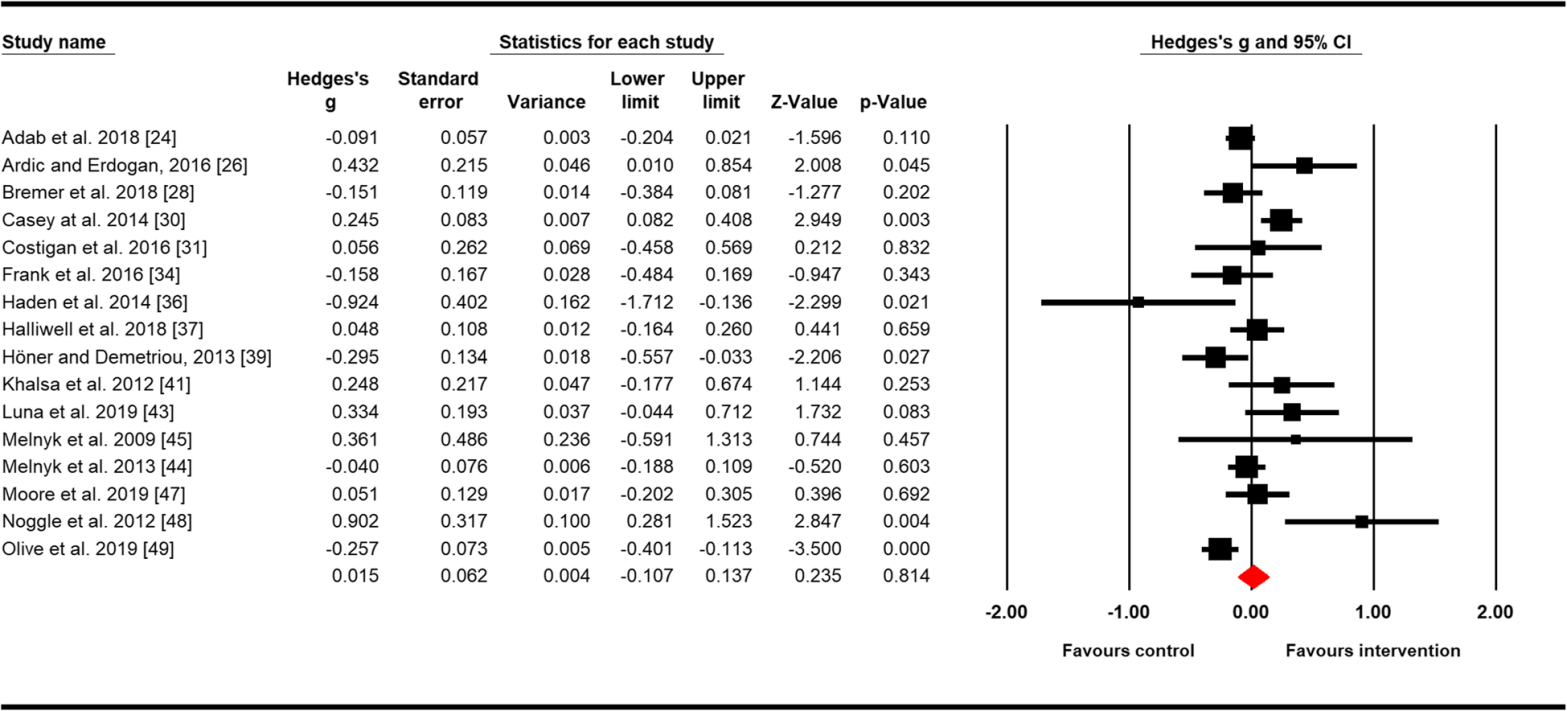
The effects of physical activity interventions in school on internalising mental health problems. Horizontal lines represent standardised mean difference (Hedges’ *g*) and 95% CIs. The diamond represents the overall estimated effect. The size of the box represents the weight of each study

**Fig. 3:**
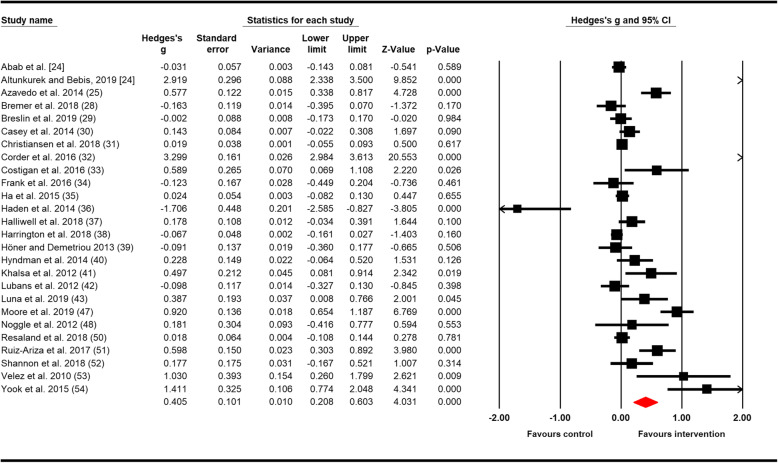
The effects of physical activity interventions in school on positive mental health. Horizontal lines represent standardised mean difference (Hedges’ *g*) and 95% CIs. The diamond represents the overall estimated effect. The size of the box represents the weight of each study

Results of the eleven meta-analyses are shown in Table 3. The number of studies included in each meta-analysis ranged from 4 for resilience to 26 for positive mental health. Figure 2 shows the forest plot of the composite outcome internalising mental health problems, and Fig. 3 for positive mental health.

**Table 3:** Meta-analysis

Outcome	No. studies	Length of INT (Weeks)	Sample size (***N***): INT + CONT at follow-up	Mean age^**a**^	Female^**b**^ (%)	Summary effect				Heterogeneity			
						Hedges’ ***g***	SE	95% CI	***p*** value	***Q***	Df (***Q***)	***p*** value	***I***^**2**^ (%)
Depressive symptoms	6	9–52	1703	12.09	49	− 0.006	0.101	− 0.204; 0.193	0.954	12.394	5	0.030	59
Anxiety	6	6–15	1060	14.79	51	0.347	0.140	0.072; 0.623	0.013	13.739	5	0.017	64
Emotional problems	5	8–52	2654	9.43	60	− 0.038	0.091	− 0.217; 0.141	0.678	17.468	4	0.002	77
Well-being	10	4–52	4565	11.18	52	0.877	0.266	0.356; 1.398	0.001	553.337	9	< 0.001	98
Health-related quality of life	11	4–52	7387	10.44	66	0.085	0.048	− 0.010; 0.179	0.078	33.983	10	< 0.001	71
Self-esteem, self-worth	10	8–52	5869	12.79	67	0.107	0.102	− 0.092; 0.307	0.292	76.414	9	< 0.001	88
Resilience	4	8–11	437	14.16	49	0.748	0.215	0.326; 1.170	0.001	10.478	3	0.015	71
Positive effect	5	4–18	676	11.10	50	0.055	0.079	− 0.100; 0.211	0.486	4.083	4	0.395	2
Negative effect	5	4–18	676	11.10	50	− 0.318	0.500	− 1.298; 0.662	0.525	53.24	3	< 0.001	94
Internalising problems	16	4–52	5045	10.21	55	0.015	0.062	− 0.107; 0.137	0.814	52.289	15	< 0.001	71
Positive mental health	26	4–52	12565	10.77	61	0.405	0.101	0.208; 0.603	< 0.001	637.615	25	< 0.001	96

^a^Mean age was calculated based on the baseline age and weighted by total sample size reported by each study.

^b^% females calculated based on entire sample size

Of the eleven outcomes measured, the effect of physical activity was significant (beneficial) for four outcomes, anxiety (Hedges’ *g* = 0.347, 95% CI = 0.072; 0.623, *p* = 0.013), resilience (Hedges’ *g* = 0.748, 95% CI = 0.326; 1.170, *p* = 0.001), well-being (Hedges’ *g* = 0.877, 95% CI = 0.356; 1.398, *p* = 0.001) and the composite outcome positive mental health (Hedges’ *g* = 0.405, 95% CI = 0.208; 0.603, *p* < 0.001). For the remaining outcomes, the meta-analysis showed no evidence of significant pooled effects of the interventions compared to controls (Table 3). Significant *Q* statistic and *I*^2^ between 59% and 98% indicated moderate to very high heterogeneity across results for all outcomes. An exception was the results for positive effect, where heterogeneity was low (*I*^2^ = 2%).

### Moderator analysis

Several potential moderators were analysed narratively for their effect on the outcomes for which more than 10 studies were included: internalising mental health problems, positive mental health, self-esteem, well-being and HRQOL. Outcome for each study was tabled (not shown) as significant negative effect, no effect or significant positive effect. Interventions were divided into the four types ‘body’, ‘body-education’ ‘body-mind’ and ‘body-education-mind’ (Table 2). The control groups could be divided into three categories: PE as usual, waitlist control, other physical activity or other activity but not physical. Other factors included in this analysis were sex distribution of the target group, age group (≤ 12 years or > 12 years), socioeconomic status (low, mixed, high), level of implementation reach (low, medium, high) and study quality (low, medium, high). Two factors showed a pattern for the outcome internalising mental health problems. One was age, where interventions in younger children showed a significantly negative or no effect and those in older children showed a significant positive or no effect. Negative effects on younger children were found in three studies [36, 39, 49]. One involved ashtanga-informed yoga three times per week for 12 weeks which led to significantly lower global self-worth and more internalising mental health problems compared to the control group [36]. Another intervention containing weekly 90-min health-promotion PE lessons consisting of strength and endurance training led to a significantly higher level of emotional problems [39] compared to the control group. The third study [49] involved specialist-taught physical education classes which led to significant higher level for depression compared to the control group. A common pattern for the three studies [36, 39, 49] with negative effects on internalising mental problems was that they all addressed younger children, and all had high implementation reach, moderate quality and a control group that received PE as usual with the same frequency and duration as the intervention group. For implementation reach, the studies with a high reach showed a significant negative or no effect on internalising mental health problems, and those with a low level of implementation showed no or a positive effect. No moderator pattern was identified for the outcomes self-esteem, well-being or positive mental health.

### Effects of publication bias across studies

Evidence for risk of publication bias was found in the meta-analysis for depressive symptoms (Egger’s *p* value = 0.024), anxiety (Egger’s *p* value = 0.045), well-being (Egger’s *p* value = 0.040), health-related quality of life (Egger’s *p* value = 0.029) and positive mental health (Egger’s *p* value = 0.022) but not for the other outcomes (Table 4). Nevertheless, publication bias did not appear to effect the conclusion about the effects of physical activity in school on these five outcomes. For anxiety, well-being, health-related quality of life and positive mental health, the corrected standardised differences in means (Hedges’ *g*) were unchanged after adjustment by the random effect trim and fill method. For depression, adjustment by the random effect trim and fill method changed the corrected standardised difference in means (Hedges’ *g*) from − 0.006 to − 0.0131, and the association remained non-significant (Hedges’ *g* adjusted 95% CI = − 0.330; 0.068). It should be noted that the power of statistical tests, especially Egger’s test, was low due to the small number of included studies, as shown by the wide confidence intervals.

**Table 4:** Analysis of publication bias

Outcome	No. studies	Egger’s test				Duval and Tweedie’s Trim and Fill^**a**^				
						Studies trimmed	Observed effect size		Adjusted effect size	
		***β***	SE	95% CI	***p*** value		Hedges ***g***	95% CI	Hedges ***g***	95% CI
Depressive symptoms	6	2.231	0.628	0.487; 3.976	0.024	3	− 0.006	− 0.204; 0.193	− 0131	− 0.330; 0.068
Anxiety	6	2.260	0.782	− 0.089; 4.430	0.045	0	0.347	0.072; 0.623	0.347	0.072; 0.623
Emotional problems	5	− 0.620	3.194	− 10.786; 9.546	0.858	0	− 0.038	− 0.217; 0.141	− 0.038	− 0.217; 0.141
Well-being	10	9.511	3.873	0.581; 18.441	0.040	0	0.877	0.356; 1.398	0.877	0.356; 1.398
Health-related quality of life	11	2.761	1.067	0.347; 5.175	0.029	0	0.084	− 0.010; 0.179	0.084	− 0.010; 0.179
Self-esteem, self-worth	10	0.727	1.503	− 2.740; 4.194	0.641	0	0.107	− 0.092; 0.307	0.107	− 0.092; 0.307
Resilience	4	− 1.005	3.218	− 14.850; 12.841	0.784	0	0.748	0.326; 1.170	0.748	0.326; 1.170
Positive effect	5	− 1.411	0.999	− 4.591; 1.768	0.253	0	0.055	− 0.100; 0.211	0.055	− 0.100; 0.211
Negative effect	5	− 2.158	4.660	− 22.210; 17.93	0.689	1	− 0.325	− 1.316; 0.665	0.114	− 2.225; 0.279
Internalising problems	16	1.114	0.921	− 0.860; 3.089	0.246	3	0.015	− 0.107; 0.137	− 0.041	− 0.167; 0.085
Positive mental health	26	3.915	1.633	0.606; 7.224	0.022	0	0.405	0.208; 0.603	0.405	0.208; 0.603

^a^Filling looks for missing studies to the left of mean

## Discussion

### Main results

To our knowledge, this is the first systematic review of school-based physical activity and sedentary behaviour interventions for children and adolescents in the general population, with self-reported mental health as the outcome. In total, 31 articles, describing 30 interventions were included. None of the included interventions were intended primarily to reduce sedentary behaviour. Out of eleven studied outcomes, we found beneficial effects of the interventions on positive mental health (Hedges’ *g* = 0.405), anxiety (Hedges’ *g* = 0.347), well-being (Hedges’ *g* = 0.877) and resilience (Hedges’ *g* = 0.748).

### Relevance of results

The results of the current review are encouraging, since school-based interventions can be delivered to all children without costs being incurred by families. Such interventions are also shown to have numerous other cardio-metabolic health benefits, especially in high-risk youngsters with obesity or high blood pressure [55, 56]. A recent systematic review, not limited to the school context or to intervention studies, also concluded that physical activity has a beneficial role in mental health in pre-schoolers, school children and adolescents [14], but with smaller effects sizes than in the present review. However, this review had some weaknesses as only two of the intervention studies included in our review had been identified. Furthermore, the authors included multiple outcomes from the same study in the meta-analysis, which assigned too much weight to those studies and decreased heterogeneity considerably [21]. Therefore, their estimates should be interpreted with caution.

If the increase in mental health problems among children and youth is partly caused by increased school stress, as suggested in a newly published report from Sweden [5], this increases the pressure on schools to implement evidence-based initiatives to halt or reverse this negative trend. The results from this review are therefore very encouraging, because they indicate that schools can counteract this development by implementing initiatives to increase physical activity during the school day. However, the present results should be interpreted with caution as the number of studies for some outcomes was relatively small, and the meta-analyses showed high heterogeneity. The fact that some studies, using relative intensive interventions, reported negative effects in younger children, points out the importance of monitoring mental health when introducing school-related physical activity interventions. This variation between results could be explained by at least three factors. First, the interventions themselves varied considerably in terms of content, duration, frequency and intensity. Some studies also combined physical and other activities, making it difficult to disentangle effects of different programme components. Second, reporting of the implementation of the interventions was very heterogeneous or absent. Taken together, we noted a large variation in fidelity and the only implementation factor we could compare between studies was reach. This means that differential effects of the interventions could also depend on how well they were implemented. Implementation of physical activity and other behavioural interventions in schools is a well-known challenge [57] and deserves greater attention and standardisation in future studies. Third, the control groups were mostly not inactive but frequently performed other activities; a design problem also shown to reduce the magnitude of effect sizes in studies of exercise for depression in adult populations [58, 59]. Therefore, as the narrative moderator analysis showed, it is not possible to recommend one specific intervention over the other. On balance, however, the results support previous findings that physical activity interventions implemented in diverse contexts have benefits for school-aged children and adolescents [14, 17].

For internalising mental health problems, age appeared to moderate programme effects, with older children over the age of 12 years experiencing favourable or no effects and younger children experiencing negative or no effects. Considering that the average age of onset for anxiety disorders is 11 years [60] and 11–13 years for depressive disorders [61], prevention effectiveness may vary depending on not only the type of intervention, but also the age or developmental stage of the child [7]. Except for age, we found no systematic pattern in effectiveness regarding the type of intervention, sex of the participants, socioeconomic status, implementation reach, study quality or the type of control group on internalising mental health problems. More studies are needed to investigate the influence of these variables on programme outcomes. For positive mental health, no systematic pattern was found regarding potential effect moderators.

Previous reviews examining the effect of different physical activity interventions on anxiety and depression in children and youth have shown varying results [7, 13]. In a systematic review from 2006, Larun et al. [13] examined the effect of exercise in prevention and treatment of anxiety and depression among children and young people and reported a statistically significant difference for depression but not for anxiety. Only one of the included studies in the review by Bonhauser et al. [62] from 2005 would have qualified for the present review but was excluded due to year of publication (i.e. before 2009). In this cRCT targeting 15-year old school children with an intervention involving extra physical exercise compared to PE as usual, a significant beneficial effect was reported on anxiety, but not on depression. The authors concluded that a school-based programme to improve physical activity in adolescents of low socioeconomic status achieved significant benefits in terms of physical fitness and mental health. This study supports our findings that physical activity in the school setting can reduce anxiety but not depression in adolescents.

Few previous reviews have investigated the effect of physical activity interventions on positive mental health, including resilience, in general populations of school children. The concept of positive mental health is a multidimensional construct [63]. Factors that have been shown to be positively correlated to positive mental health include male sex, younger age, higher education, higher income and social relations [64]. Barry et al. [64] notes that the concept is connected to socio-cultural norms. Resilience refers to a dynamic process encompassing positive adaptation within the context of significant adversity [65]. The beneficial effects on resilience in our review suggest that physical activity interventions in the school context may be important to help children cope with adversities. However, the result is based on only four studies and more research is therefore needed in this area. A review by Khalsa et al. [66] investigating the effect of yoga interventions in the school context on mental, emotional, physical and behavioural health characteristics concluded that yoga is a potentially effective strategy to improve child health in the school setting. The review included 47 yoga studies with different study designs. Like our review, the included studies were heterogeneous in terms of duration and frequency.

In order to deliver successful interventions, it is important to know by which mechanisms physical activity is leading to changes in mental health. Based on the literature, Lubans et al. [67] developed a conceptual model for the effects of physical activity on mental health by three mechanisms: neurobiological, psychosocial and behavioural (e.g. by improving sleep). In our review, we only analysed self-reported psychosocial outcomes as indicators of mental health, which are also the most commonly reported. However, it is possible that some interventions may work through the other two mechanisms to improve mental health. As emphasised by Lubans et al. [67], improving our understanding of the mechanisms of how physical activity leads to better mental health may assist in the development of more specific and effective interventions.

### Strengths and limitations of the review

The review has several strengths such as the comprehensive literature search in nine databases, pre-registration of the study protocol in the Prospero database, and that the search, data extraction and quality assessment was done by two researchers independently. The review also has some limitations. Although we searched 9 databases, we might have missed some relevant articles in other languages than English. The interventions varied considerably as did the control groups resulting in high heterogeneity in effect sizes. The selection of instruments for each outcome and prioritisation among instruments involved some degree of arbitrariness, which led to slightly different pooled effect sizes depending on which instruments were included. Other investigators do not always describe which instruments are included under each outcome. We decided to include this information in Online resource 4 for transparency reasons. More research is required to reach consensus in the research community regarding how to combine instruments under different outcomes for meta-analytic purposes. The included studies were also of mixed methodological quality, and several of them were underpowered. Moreover, for pragmatic reasons we decided to exclude studies which solely included broader aspects of positive mental health and internalising mental health problems. We could thus have overlooked important findings. New studies are under way [68–71] and results can be expected within a few years. These may show whether the current findings can be confirmed and, if so, what type of interventions give the best effects.

## Conclusions

The results of this systematic review indicate that school-related interventions aiming to promote physical activity can reduce anxiety, increase resilience, increase well-being and improve positive mental health of children and young people. Considering the positive effects of physical activity on health in general, these findings may reinforce school-based initiatives to increase physical activity. Future studies should more clearly describe the activities of the control group and whether the activity is added to or replacing ordinary physical education lessons in order to aid the interpretation of results. Our findings also highlight the need for more high-quality universal physical activity interventions in the school context and standardised reporting of implementation. To further understand how such interventions work and can be used in practice, there is a need to focus on mechanisms of action and on evaluation of the implementation process.

## Supplementary information

**Additional file 1.** Online resource 1. Search strategy.

**Additional file 2.** Online resource 2. Excluded studies.

**Additional file 3.** Online resource 3. Implementation factors.

**Additional file 4.** Online resource 4. Outcomes and instruments.

**Additional file 5.** Online resource 5. Quality assessment.

## Data Availability

All data generated or analysed during this study are included in this published article and its supplementary information files.

## Abbreviations

CI: Confidence interval
cRCT: Cluster-RCTs
EPHPP: Effective Public Health Practice Project Quality Assessment Tool
RCT: Randomised controlled trials
PE: Physical education
SD: Standard deviation
SE: Standard error
SMD: Standardised mean difference
