# Supplementary material for: School-related physical activity interventions and mental health among children: a systematic review and meta-analysis
Source: Sports Med Open. 2020 Jun 16;6:25. doi: 10.1186/s40798-020-00254-x (PMC7297899; doi:10.1186/s40798-020-00254-x)
Supplement: Supplementary file 1 — Additional file 1. Online resource 1. Search strategy. [file 40798_2020_254_MOESM1_ESM.pdf]

Karolinska Institutet

Documentation of search strategies
University Library search consultation group

Date: Update October 2019 (Original search March 2018)

Topic/research question: School related physical activity/sedentary behavior and mental health in children

Name of researcher(s): Susanne Andermo & Liselotte Schäfer Elinder, PHS

Librarian(s): Klas Moberg & Carl Gornitzki

Databases:

1. Medline , Epub Ahead of Print, In-Process & Other Non-Indexed Citations, Ovid MEDLINE(R) Daily and Ovid MEDLINE(R) (Ovid)
2. Psycinfo (Ovid)
3. Web of Science Core Collection
4. ERIC (Proquest)
5. Sociological Abstracts (ProQuest)

Total number of hits:

- Before deduplication: 14 821 (Original search 11 372)
- After deduplication: 2584 (Original search 7673), after deduplication against the original search

---

Comments:

PRISMA 2009 Flow Diagram[1]

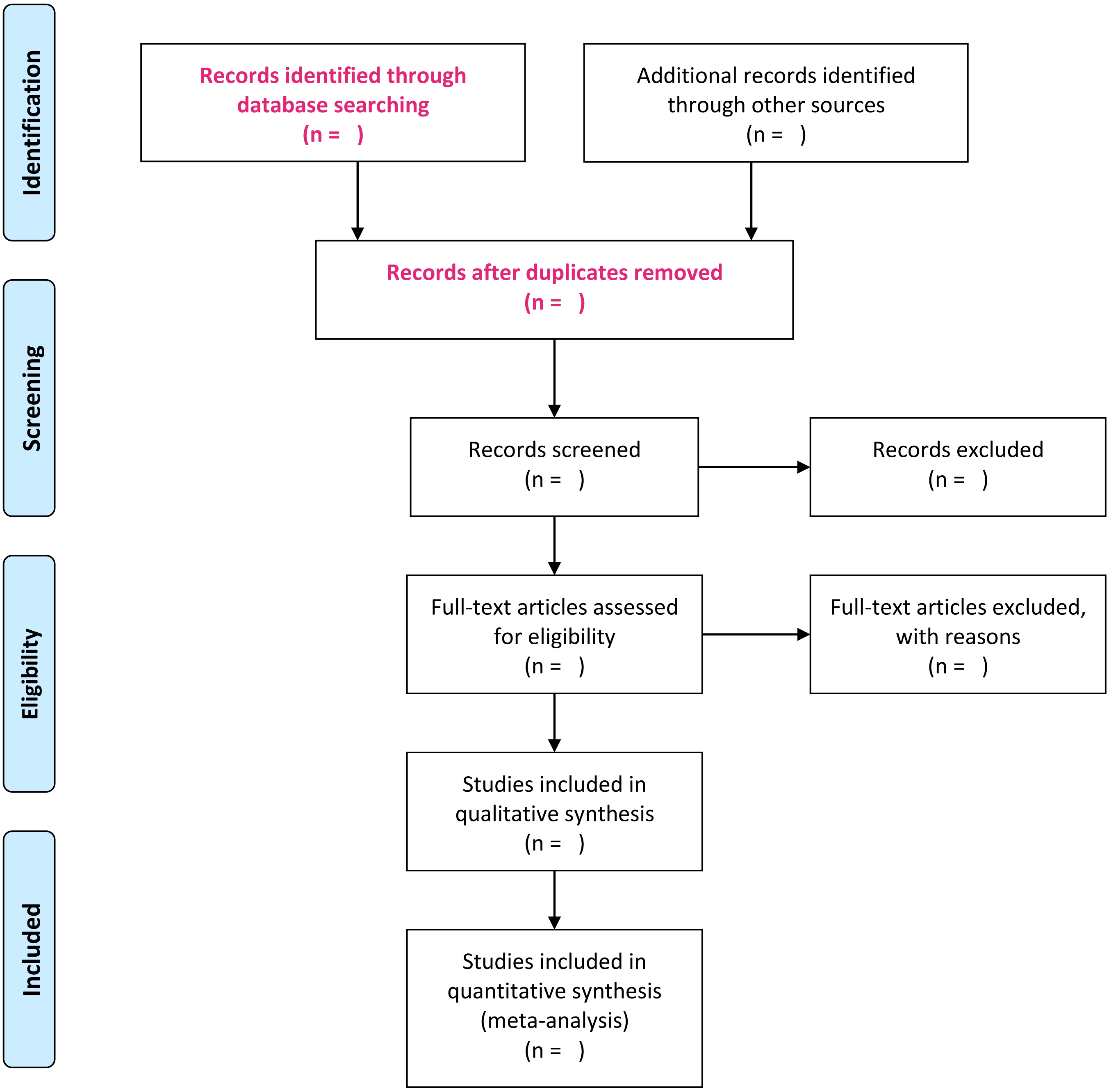

[1] From: Moher D, Liberati A, Tetzlaff J, Altman DG, The PRISMA Group (2009). Preferred Reporting Items for Systematic Reviews and Meta-Analyses: The PRISMA Statement. PLoS Med 6(6): e1000097. doi:10.1371/journal.pmed1000097. For more information, visit www.prisma-statement.org.

Karolinska Institutet

1. Medline, Epub Ahead of Print, In-Process & Other Non-Indexed Citations, Ovid MEDLINE(R) Daily and Ovid MEDLINE(R) (Ovid)

Date of Search: 24 October 2019<br><br>Number of hits: 4546 (original search 3474)<br><br>Comments:	Field labels:<br><br>• .ti,ab.= titel & abstract<br>• .kf. = keywords<br>• / = MeSH<br>• exp/ = MeSH, exploded<br>• adj = the terms are adjacent with no words in between in the order presented<br>• adjx = adjacent within x words<br>• * = truncation of word for alternate endings<br>• $ = truncation of word for alternate endings

1. Schools/
2. School Health Services/
3. (school* or highschool* or grade-6 or grade-six or grade-7 or grade-seven or grade-8 or grade-eight or grade-9 or grade-nine or grade-10 or grade-11 or grade-12 or student* or pupil*).ti,ab,kf.
4. or/1-3

5. Mental Disorders/
6. Mental Health/
7. Psychopathology/
8. Child Welfare/
9. Child Psychiatry/
10. Adolescent Psychiatry/
11. Adjustment Disorders/
12. Affective Symptoms/
13. exp Mood Disorders/
14. Depression/
15. exp Anxiety Disorders/
16. Anxiety/
17. Fear/
18. Panic/
19. Performance Anxiety/
20. Compulsive Personality Disorder/
21. Obsessive Behavior/
22. Compulsive Behavior/

23. Quality of life/
24. Personal satisfaction/
25. Happiness/
26. Pessimism/
27. Self Concept/
28. Body Image/
29. Self Efficacy/
30. Sense of Coherence/
31. Adaptation, Psychological/
32. Resilience, Psychological/
33. ((mental or emotional) adj3 (health or disorder* or disease* or problem* or symptom* or illness* or ill health or illhealth or distress or instabilit*)).ti,ab,kf.
34. ((psychiatric or psychologic) adj3 (problem* or symptom* or disorder* or ill health or illhealth or distress or illness*)).ti,ab,kf.
35. (psychopathology or psychosocial or abnormal psychology or internal distress or internalising or internalizing or adjustment disorder* or affective symptom* or affective problem* or affective distress or mood or moods or affective disorde* or affective syndrome* or depress* or dysthymi* or sad or sadness or unhapp* or hopeless* or loneliness or lonely or worries or worry or social withdrawal or phobia or phobic or claustrophobia or agoraphob* or ophidiophobia or school phobia or neophobia or avoidant disorder* or overanxious or nervousness or panic disorder* or panic attack* or compulsive or compulsion or compulsiveness or obsess* or anankastic or intrusive thoughts or intrusive thinking or fear or avoidance or pessimism or pessimistic or despondency or despair or frustrat* or anxiety or anxious).ti,ab,kf.
36. (well-being or wellbeing or wellness or optimis* or cheerful* or contentment or elated or elation or joy or enjoyment or good feeling* or good mood or happiness or happy or satisfaction or quality of life or HRQoL or QoL or sense of coherence or resilience or coping).ti,ab,kf.
37. (positive adj3 (affect* or emotion* or mood*)).ti,ab,kf.
38. (self adj3 (concept* or perception* or acceptance or confidence or esteem* or image or efficacy or reliance or worth or compassion)).ti,ab,kf.
39. or/5-38

40. "Physical Education and Training"/
41. Physical Fitness/
42. exp Exercise/
43. exp Motor Activity/
44. Leisure Activities/
45. Sedentary Lifestyle/
46. exp Sports/
47. exp Exercise Movement Techniques/
48. exp "Play and Playthings"/
49. Movement/
50. exp Television/
51. exp Internet/
52. Computers/
53. (physical* adj2 (fitness or education or condition or mobility or activ* or effort*)).ti,ab,kf.
54. (activ* adj3 (commut* or pause* or break* or lesson* or recess* or transport* or travel* or play* or movement* or lifestyle or locomotor or leisure or extracurricular or extra-curricular)).ti,ab,kf.
55. (aerobic* or aikido or archery or athletics or badminton or ballgame* or ball game* or baseball or basketball or biathl* or bicycle* or bicycling or bike or biking or bowling or boxing or calisthenic* or

cardiopulmonary conditioning or callisthenic* or canoe* or cricket or curling or cycling or danc* or diving or exercise* or fencing or floorball or floor ball or football or golf* or jogging or jumping or handball or hopping or gigong or gi gong or gymnastic* or hiit or hockey or horseback riding or horse riding or isometric climbing or jogging or judo or jujitsu or karate or kung fu or kung or marathon* or martial art* or mountaineer* or neuromuscular facilitation* or power lifting or pilates or plyometric* or racquetball or racketball or racket ball or rowing or rugby or running or sail* or skateboard* or skating or skiing or snowboard* or soccer or softball or squash or sport* or stretching or plyometric* or swim* or tae kwon do or taekwondo or taiji or taijiquan or taichi or walk* or warm-up or yoga or multidisciplinary rehabilitation or sport* or relaxation* or volleyball or walking or weight* lifting or lifting weight* or weightlifting or isometric climbing or wrestling).ti,ab,kf.
56. sedentary.ti,ab,kf.
57. (inactiv* or sitting or stationary or TV or television).ti,ab,kf.
58. ((screen or media or computer or video or internet or web or outdoor) adj3 time).ti,ab,kf.
59. ((media or computer or video or internet or electronic or web or outdoor) adj3 (game* or gaming or play*)).ti,ab,kf.
60. ((media or computer or video or internet or web or smart phone or smartphone) adj1 "use").ti,ab,kf.
61. or/40-60

62. 4 and 39 and 61

63. limit 62 to yr="2009 -Current"

64. limit 63 to (english or swedish)

65. randomized controlled trial.pt.
66. controlled clinical trial.pt.
67. random*.ab.
68. placebo.ab.
69. clinical trials as topic.sh.
70. trial.ti.
71. Observational Study/
72. Epidemiologic studies/
73. Cross-Sectional Studies/
74. exp case control studies/
75. exp cohort studies/
76. Non-Randomized Controlled Trials as Topic/
77. Case control.ti,ab,kf.
78. (cohort adj (study or studies)).ti,ab,kf.
79. Cohort analy$.ti,ab,kf.
80. ((Follow up or Followup) adj (study or studies)).ti,ab,kf.
81. (observational adj (study or studies)).ti,ab,kf.
82. Longitudinal.ti,ab,kf.
83. Retrospective.ti,ab,kf.
84. Prospective.ti,ab,kf.
85. Cross sectional.ti,ab,kf.
86. Quasi-Experimental.ti,ab,kf.
87. Health Policy/
88. Politics/
89. Policy Making/

Karolinska Institutet

90. Healthy People Programs/
91. (policy or policies or politic* or action plan* or regulat*).ti,ab,kf.
92. or/65-91

93. 64 and 92

2. Psycinfo (Ovid)

Date of Search: 24 October 2019<br><br>Number of hits: 2898 (original search 2464)<br><br>Comments:	Field labels:<br><br>• .ti,ab.= titel & abstract<br>• .id. = keywords<br>• / = PsycINFO Thesaurus terms<br>• exp/ = PsycINFO Thesaurus terms, exploded<br>• adj = the terms are adjacent with no words in between in the order presented<br>• adjx = adjacent within x words<br>• * = truncation of word for alternate endings<br>• $ = truncation of word for alternate endings

1. schools/
2. boarding schools/
3. charter schools/
4. elementary schools/
5. high schools/
6. junior high schools/
7. middle schools/
8. nongraded schools/
9. nursery schools/
10. school environment/
11. classroom environment/
12. school facilities/
13. (school* or highschool* or grade-6 or grade-six or grade-7 or grade-seven or grade-8 or grade-eight or grade-9 or grade-nine or grade-10 or grade-11 or grade-12 or student* or pupil*).ti,ab,id.
14. or/1-13

15. mental disorders/
16. mental health/
17. exp psychopathology/
18. child welfare/
19. well being/
20. child psychiatry/
21. adolescent psychiatry/
22. adjustment disorders/
23. exp affective disorders/
24. "depression (emotion)"/
25. exp anxiety disorders/
26. exp anxiety/
27. fear/
28. panic/
29. panic attack/

30. obsessions/
31. exp compulsions/
32. "quality of life"/
33. satisfaction/
34. life satisfaction/
35. happiness/
36. pessimism/
37. self-concept/
38. self-confidence/
39. self-esteem/
40. self-perception/
41. exp body image/
42. self-efficacy/
43. "sense of coherence"/
44. coping behavior/
45. "resilience (psychological)"/
46. ((mental or emotional) adj3 (health or disorder* or disease* or problem* or symptom* or illness* or ill health or illhealth or distress or instabilit*)).ti,ab,id.
47. ((psychiatric or psychologic) adj3 (problem* or symptom* or disorder* or ill health or illhealth or distress or illness*)).ti,ab,id.
48. (psychopathology or psychosocial or abnormal psychology or internal distress or internalising or internalizing or adjustment disorder* or affective symptom* or affective problem* or affective distress or mood or moods or affective disorde* or affective syndrome* or depress* or dysthymi* or sad or sadness or unhapp* or hopeless* or loneliness or lonely or worries or worry or social withdrawal or phobia or phobic or claustrophobia or agoraphob* or ophidiophobia or school phobia or neophobia or avoidant disorder* or overanxious or nervousness or panic disorder* or panic attack* or compulsive or compulsion or compulsiveness or obsess* or anankastic or intrusive thoughts or intrusive thinking or fear or avoidance or pessimism or pessimistic or despondency or despair or frustrat* or anxiety or anxious).ti,ab,id.
49. (well-being or wellbeing or wellness or optimis* or cheerful* or contentment or elated or elation or joy or enjoyment or good feeling* or good mood or happiness or happy or satisfaction or quality of life or HRQoL or QoL or sense of coherence or resilience or coping).ti,ab,id.
50. (positive adj3 (affect* or emotion* or mood*)).ti,ab,id.
51. (self adj3 (concept* or perception* or acceptance or confidence or esteem* or image or efficacy or reliance or worth or compassion)).ti,ab,id.
52. or/15-51

53. physical education/
54. physical fitness/
55. exp exercise/
56. physical activity/
57. exp recreation/
58. exp sports/
59. games/
60. computer games/
61. television/
62. screen time/
63. sedentary behavior/
64. exp internet/
65. exp social media/
66. internet usage/
67. computers/
68. (physical* adj2 (fitness or education or condition or mobility or activ* or effort*)).ti,ab,id.
69. (activ* adj3 (commut* or pause* or break* or lesson* or recess* or transport* or travel* or play* or movement* or lifestyle or locomotor or leisure or extracurricular or extra-curricular)).ti,ab,id.
70. (aerobic* or aikido or archery or athletics or badminton or ballgame* or ball game* or baseball or basketball or biathl* or bicycle* or bicycling or bike or biking or bowling or boxing or calisthenic* or cardiopulmonary conditioning or callisthenic* or canoe* or cricket or curling or cycling or danc* or diving or

Karolinska Institutet

exercise* or fencing or floorball or floor ball or football or golf* or jogging or jumping or handball or hopping or gigong or gi gong or gymnastic* or hiit or hockey or horseback riding or horse riding or isometric climbing or jogging or judo or jujitsu or karate or kung fu or kung or marathon* or martial art* or mountaineer* or neuromuscular facilitation* or power lifting or pilates or plyometric* or racquetball or racketball or racket ball or rowing or rugby or running or sail* or skateboard* or skating or skiing or snowboard* or soccer or softball or squash or sport* or stretching or plyometric* or swim* or tae kwon do or taekwondo or taiji or taijiquan or taichi or walk* or warm-up or yoga or multidisciplinary rehabilitation or sport* or relaxation* or volleyball or walking or weight* lifting or lifting weight* or weightlifting or isometric climbing or wrestling).ti,ab,id.
71. sedentary.ti,ab,id.
72. (inactiv* or sitting or stationary or TV or television).ti,ab,id.
73. ((screen or media or computer or video or internet or web or outdoor) adj3 time).ti,ab,id.
74. ((media or computer or video or internet or electronic or web or outdoor) adj3 (game* or gaming or play*)).ti,ab,id.
75. ((media or computer or video or internet or web or smart phone or smartphone) adj1 "use").ti,ab,id.
76. or/53-75

77. 14 and 52 and 76

78. limit 77 to yr="2009 -Current"

79. limit 78 to (english or swedish)

80. clinical trials/
81. random*.ab.
82. placebo.ab.
83. trial.ti.
84. retrospective studies/
85. followup studies/
86. exp longitudinal studies/
87. quasi experimental methods/
88. observation methods/
89. Case control.ti,ab,id.
90. (cohort adj (study or studies)).ti,ab,id.
91. Cohort analy$.ti,ab,id.
92. ((Follow up or Followup) adj (study or studies)).ti,ab,id.
93. (observational adj (study or studies)).ti,ab,id.
94. Longitudinal.ti,ab,id.
95. Retrospective.ti,ab,id.
96. Prospective.ti,ab,id.
97. Cross sectional.ti,ab,id.
98. Quasi-Experimental.ti,ab,id.
99. health care policy/
100. government policy making/
101. policy making/
102. politics/
103. (policy or policies or politic* or action plan* or regulat*).ti,ab,id.
104. or/80-103

105. 79 and 104

3. Web of Science Core Collection

Date of Search: 24 October 2019<br>Number of hits: 5442 (original search 4099)<br>Comments:	Field labels:<br>• TS = Topic = title, abstract & keyword<br>• NEAR/x = adjacent within x words

TOPIC: (school* or highschool* or "grade-6" or "grade-six" or "grade-7" or "grade-seven" or "grade-8" or "grade-eight" or "grade-9" or "grade-nine" or "grade-10" or "grade-11" or "grade-12" or student* or pupil*)

AND

TOPIC: (("mental" or "emotional") NEAR/3 ("health" or disorder* or disease* or problem* or symptom* or illness* or "ill health" or "illhealth" or "distress" or instabilit*)) *OR* **TOPIC:** (("psychiatric" or "psychologic") NEAR/3 (problem* or symptom* or disorder* or "ill health" or "illhealth" or "distress" or illness*)) *OR* **TOPIC:** ("psychopathology" or "psychosocial" or "abnormal psychology" or "internal distress" or "internalising" or "internalizing" or "adjustment disorder*" or "affective symptom*" or "affective problem*" or "affective distress" or "mood" or "moods" or "affective disorde*" or "affective syndrome*" or depress* or dysthymi* or "sad" or "sadness" or unhapp* or hopeless* or "loneliness" or "lonely" or "worries" or "worry" or "social withdrawal" or "phobia" or "phobic" or "claustrophobia" or agoraphob* or "ophidiophobia" or "school phobia" or "neophobia" or "avoidant disorder*" or "overanxious" or "nervousness" or "panic disorder*" or "panic attack*" or "compulsive" or "compulsion" or "compulsiveness" or obsess* or anankastic or "intrusive thoughts" or "intrusive thinking" or "fear" or "avoidance" or "pessimism" or "pessimistic" or "despondency" or "despair" or frustrat* or "anxiety" or "anxious") *OR* **TOPIC:** ("well-being" or "wellbeing" or "wellness" or optimis* or cheerful* or "contentment" or "elated" or "elation" or "joy" or "enjoyment" or "good feeling*" or "good mood" or "happiness" or "happy" or "satisfaction" or "quality of life" or "HRQoL" or "QoL" or "sense of coherence" or "resilience" or "coping") *OR* **TOPIC:** ("positive" adj3 (affect* or emotion* or mood*)) *OR* **TOPIC:** ("self" adj3 (concept* or perception* or "acceptance" or "confidence" or esteem* or "image" or "efficacy" or "reliance" or "worth" or "compassion"))

AND

TOPIC: (physical* NEAR/2 ("fitness" or "education" or "condition" or "mobility" or activ* or effort*)) *OR* **TOPIC:** (activ* NEAR/3 (commut* or pause* or break* or lesson* or recess* or transport* or travel* or play* or movement* or "lifestyle" or "locomotor" or "leisure" or "extracurricular" or "extra-curricular")) *OR* **TOPIC:** (aerobic* or "aikido" or "archery" or "athletics" or "badminton" or ballgame* or "ball game*" or "baseball" or "basketball" or biathl* or bicycle* or "bicycling" or "bike" or "biking" or "bowling" or "boxing" or calisthenic* or "cardiopulmonary conditioning" or callisthenic* or canoe* or "cricket" or "curling" or "cycling" or danc* or "diving" or exercise* or "fencing" or "floorball" or "floor ball" or "football" or golf* or "jogging" or "jumping" or "handball" or "hopping" or "gigong" or "gi gong" or gymnastic* or "hiit" or "hockey" or "horseback riding" or "horse riding" or "isometric climbing" or "jogging" or "judo" or "jujitsu" or "karate" or "kung fu" or "kung" or marathon* or "martial art*" or mountaineer* or "neuromuscular facilitation*" or "power

lifting" or "pilates" or plyometric* or "racquetball" or "racketball" or "racket ball" or "rowing" or "rugby" or "running" or sail* or skateboard* or "skating" or "skiing" or snowboard* or "soccer" or "softball" or "squash" or sport* or "stretching" or plyometric* or swim* or "tae kwon do" or "taekwondo" or "taiji" or "taijiquan" or "taichi" or walk* or "warm-up" or "yoga" or "multidisciplinary rehabilitation" or sport* or relaxation* or "volleyball" or "walking" or "weight* lifting" or "lifting weight*" or "weightlifting" or "isometric climbing" or "wrestling" or "sedentary" or inactiv* or "sitting" or "stationary" or "TV" or "television") *OR* **TOPIC:** (("screen" or "media" or "computer" or "video" or "internet" or "web" or "outdoor") NEAR/3 "time") *OR* **TOPIC:** (("media" or "computer" or "video" or "internet" or "electronic" or "web" or "outdoor") NEAR/3 (game* or "gaming" or play*)) *OR* **TOPIC:** (("media" or "computer" or "video" or "internet" or "web" or "smart phone" or "smartphone") NEAR/1 "use")

AND

TOPIC: (random* or "placebo" or "Case control" or "cohort study" or "cohort studies" or "Cohort analy*" or "Follow up study" or "Follow up studies" or "Followup study" or "Followup studies" or "observational study" or "observational studies" or "Longitudinal" or "Retrospective" or "Prospective" or "Cross sectional" or "Quasi-Experimental" or "policy" or "policies" or politic* or "action plan*" or regulat*) *OR* **TITLE:** ("trial")

Refined by: LANGUAGES: (ENGLISH) (No articles in Swedish)
ESCI Timespan=2009-2019

4. ERIC (Proquest)

Date of Search: 24 October 2019<br><br>Number of hits: 1538 (original search 1040)<br><br>Comments:	Field labels:<br><br>• MAINSUBJECT.EXACT.EXPLODE = exploded controlled term<br>• MAINSUBJECT.EXACT = non exploded controlled term<br>• ti = title<br>• ab = abstract<br>• NEAR/x = within x words, regardless of order<br>• * = truncation of word for alternate endings

((MAINSUBJECT.EXACT("Schools") OR MAINSUBJECT.EXACT("Bilingual Schools") OR MAINSUBJECT.EXACT("Biracial Schools") OR MAINSUBJECT.EXACT.EXPLODE("Boarding Schools") OR MAINSUBJECT.EXACT("Community Schools") OR MAINSUBJECT.EXACT("Consolidated Schools") OR MAINSUBJECT.EXACT("Day Schools") OR MAINSUBJECT.EXACT("Disadvantaged Schools") OR MAINSUBJECT.EXACT("Elementary Schools") OR MAINSUBJECT.EXACT("Experimental Schools") OR MAINSUBJECT.EXACT("Free Schools") OR MAINSUBJECT.EXACT("International Schools") OR MAINSUBJECT.EXACT("Laboratory Schools") OR MAINSUBJECT.EXACT("Magnet Schools") OR MAINSUBJECT.EXACT("Middle Schools") OR MAINSUBJECT.EXACT("Montessori Schools") OR MAINSUBJECT.EXACT("Multiunit Schools") OR MAINSUBJECT.EXACT("Neighborhood Schools") OR MAINSUBJECT.EXACT("Nursery Schools") OR MAINSUBJECT.EXACT("Open Plan Schools") OR MAINSUBJECT.EXACT("Private Schools") OR MAINSUBJECT.EXACT.EXPLODE("Parochial Schools") OR MAINSUBJECT.EXACT("Proprietary Schools") OR MAINSUBJECT.EXACT.EXPLODE("Public Schools") OR MAINSUBJECT.EXACT("Racially Balanced Schools") OR MAINSUBJECT.EXACT("Regional Schools") OR MAINSUBJECT.EXACT("Rural Schools") OR MAINSUBJECT.EXACT.EXPLODE("Secondary Schools") OR MAINSUBJECT.EXACT("Single Sex Schools") OR MAINSUBJECT.EXACT("Slum Schools") OR MAINSUBJECT.EXACT("Small Schools") OR MAINSUBJECT.EXACT("One Teacher Schools") OR MAINSUBJECT.EXACT("Special Schools") OR MAINSUBJECT.EXACT("State Schools") OR MAINSUBJECT.EXACT("Suburban Schools") OR MAINSUBJECT.EXACT("Summer Schools") OR MAINSUBJECT.EXACT("Traditional Schools") OR MAINSUBJECT.EXACT("Urban Schools") OR MAINSUBJECT.EXACT("Year Round Schools") OR MAINSUBJECT.EXACT("Educational Environment") OR MAINSUBJECT.EXACT("Classroom Environment") OR MAINSUBJECT.EXACT("Grade 6 ") OR MAINSUBJECT.EXACT("Grade 7") OR MAINSUBJECT.EXACT("Grade 8") OR MAINSUBJECT.EXACT("Grade 9") OR MAINSUBJECT.EXACT("Grade 10") OR MAINSUBJECT.EXACT("Grade 11") OR MAINSUBJECT.EXACT("Grade 12") OR MAINSUBJECT.EXACT("School Health Services")) OR (ti(school* or highschool* or grade-6 or grade-six or grade-7 or grade-seven or grade-8 or grade-eight or grade-9 or grade-nine or grade-10 or grade-11 or grade-12 or student* or pupil*) OR ab(school* or highschool* or grade-6 or grade-six or grade-7 or grade-seven or grade-8 or grade-eight or grade-9 or grade-nine or grade-10 or grade-11 or grade-12 or student* or pupil*)))

AND

((MAINSUBJECT.EXACT("Mental Disorders") OR MAINSUBJECT.EXACT("Mental Health") OR MAINSUBJECT.EXACT("Psychopathology") OR MAINSUBJECT.EXACT("Well Being") OR MAINSUBJECT.EXACT("Child Welfare") OR MAINSUBJECT.EXACT("Child Psychology") OR MAINSUBJECT.EXACT("Coping") OR MAINSUBJECT.EXACT("Emotional Adjustment") OR MAINSUBJECT.EXACT("Adjustment (to Environment)") OR MAINSUBJECT.EXACT("Depression (Psychology)") OR MAINSUBJECT.EXACT("Anxiety Disorders") OR MAINSUBJECT.EXACT("Anxiety") OR MAINSUBJECT.EXACT("Separation Anxiety") OR MAINSUBJECT.EXACT("Fear") OR MAINSUBJECT.EXACT("School Phobia") OR MAINSUBJECT.EXACT("Quality of Life") OR MAINSUBJECT.EXACT("Satisfaction") OR MAINSUBJECT.EXACT("Life Satisfaction") OR MAINSUBJECT.EXACT("Happiness") OR MAINSUBJECT.EXACT.EXPLODE("Self Concept ") OR MAINSUBJECT.EXACT("Resilience (Psychology)")) OR (ti(((mental or emotional) NEAR/3 (health or disorder* or disease* or problem* or symptom* or illness* or "ill health" or illhealth or distress or instabilit*)) OR ((psychiatric or psychologic) NEAR/3 (problem* or symptom* or disorder* or "ill health" or illhealth or distress or illness*)) OR (psychopathology or psychosocial or "abnormal psychology" or "internal distress" or internalising or internalizing or "adjustment disorder*" or "affective symptom*" or "affective problem*" or "affective distress" or mood or moods or "affective disorde*" or "affective syndrome*" or depress* or dysthymi* or sad or sadness or unhapp* or hopeless* or loneliness or lonely or worries or worry or "social withdrawal" or phobia or phobic or claustrophobia or agoraphob* or ophidiophobia or "school phobia" or neophobia or "avoidant disorder*" or overanxious or nervousness or "panic disorder*" or "panic attack*" or compulsive or compulsion or compulsiveness or obsess* or anankastic or "intrusive thoughts" or "intrusive thinking" or fear or avoidance or pessimism or pessimistic or despondency or despair or frustrat* or anxiety or anxious) OR (well-being or wellbeing or wellness or optimis* or cheerful* or contentment or elated or elation or joy or enjoyment or "good feeling*" or "good mood" or happiness or happy or satisfaction or "quality of life" or HRQoL or QoL or "sense of coherence" or resilience or coping) OR (positive NEAR/3 (affect* or emotion* or mood*)) OR (self NEAR/3 (concept* or perception* or acceptance or confidence or esteem* or image or efficacy or reliance or worth or compassion))) OR ab(((mental or emotional) NEAR/3 (health or disorder* or disease* or problem* or symptom* or illness* or "ill health" or illhealth or distress or instabilit*)) OR ((psychiatric or psychologic) NEAR/3 (problem* or symptom* or disorder* or "ill health" or illhealth or distress or illness*)) OR (psychopathology or psychosocial or "abnormal psychology" or "internal distress" or internalising or internalizing or "adjustment disorder*" or "affective symptom*" or "affective problem*" or "affective distress" or mood or moods or "affective disorde*" or "affective syndrome*" or depress* or dysthymi* or sad or sadness or unhapp* or hopeless* or loneliness or lonely or worries or worry or "social withdrawal" or phobia or phobic or claustrophobia or agoraphob* or ophidiophobia or "school phobia" or neophobia or "avoidant disorder*" or overanxious or nervousness or "panic disorder*" or "panic attack*" or compulsive or compulsion or compulsiveness or obsess* or anankastic or "intrusive thoughts" or "intrusive thinking" or fear or avoidance or pessimism or pessimistic or despondency or despair or frustrat* or anxiety or anxious) OR (well-being or wellbeing or wellness or optimis* or cheerful* or contentment or elated or elation or joy or enjoyment or "good feeling*" or "good mood" or happiness or happy or satisfaction or "quality of life" or HRQoL or QoL or "sense of coherence" or resilience or coping) OR (positive NEAR/3 (affect* or emotion* or mood*)) OR (self NEAR/3 (concept* or perception* or acceptance or confidence or esteem* or image or efficacy or reliance or worth or compassion)))))

AND

((MAINSUBJECT.EXACT.EXPLODE("Physical Education") OR MAINSUBJECT.EXACT("Physical Education Facilities") OR MAINSUBJECT.EXACT.EXPLODE("Physical Fitness ") OR MAINSUBJECT.EXACT.EXPLODE("Physical Activities") OR MAINSUBJECT.EXACT("Physical Activity Level") OR MAINSUBJECT.EXACT("Leisure Time") OR MAINSUBJECT.EXACT("Recreation") OR MAINSUBJECT.EXACT("Recreational Activities") OR MAINSUBJECT.EXACT.EXPLODE("Recreational Facilities") OR MAINSUBJECT.EXACT("Playground Activities") OR MAINSUBJECT.EXACT.EXPLODE("Games") OR MAINSUBJECT.EXACT("Play") OR MAINSUBJECT.EXACT("Television") OR MAINSUBJECT.EXACT("Childrens Television") OR MAINSUBJECT.EXACT("Public Television") OR MAINSUBJECT.EXACT("Internet") OR MAINSUBJECT.EXACT.EXPLODE("Computers") OR MAINSUBJECT.EXACT("Recess Breaks") OR MAINSUBJECT.EXACT("Extracurricular Activities")) OR (ti((physical* NEAR/2 (fitness or education or condition or mobility or activ* or effort*)) OR (activ* NEAR/3 (commut* or pause* or break* or lesson* or recess* or transport* or travel* or play* or movement* or lifestyle or locomotor or leisure or extracurricular or extra-curricular)) OR (aerobic* or aikido or archery or athletics or badminton or ballgame* or "ball game*" or baseball or basketball or biathl* or bicycle* or bicycling or bike or biking or bowling or boxing or calisthenic* or "cardiopulmonary conditioning" or callisthenic* or canoe* or cricket or curling or cycling or danc* or diving or exercise* or fencing or floorball or "floor ball" or football or golf* or jogging or jumping or handball or hopping or gigong or "gi gong" or gymnastic* or hiit or hockey or "horseback riding" or "horse riding" or "isometric climbing" or jogging or judo or jujitsu or karate or "kung fu" or kung or marathon* or "martial art*" or mountaineer* or "neuromuscular facilitation*" or "power lifting" or pilates or plyometric* or racquetball or racketball or "racket ball" or rowing or rugby or running or sail* or skateboard* or skating or skiing or snowboard* or soccer or softball or squash or sport* or stretching or plyometric* or swim* or "tae kwon do" or taekwondo or taiji or taijiquan or taichi or walk* or warm-up or yoga or "multidisciplinary rehabilitation" or sport* or relaxation* or volleyball or walking or "weight* lifting" or "lifting weight*" or weightlifting or "isometric climbing" or wrestling or sedentary or inactiv* or sitting or stationary or TV or television) OR ((screen or media or computer or video or internet or web or outdoor) NEAR/3 time) OR ((media or computer or video or internet or electronic or web or outdoor) NEAR/3 (game* or gaming or play*)) OR ((media or computer or video or internet or web or "smart phone" or smartphone) NEAR/1 "use")) OR ab((physical* NEAR/2 (fitness or education or condition or mobility or activ* or effort*)) OR (activ* NEAR/3 (commut* or pause* or break* or lesson* or recess* or transport* or travel* or play* or movement* or lifestyle or locomotor or leisure or extracurricular or extra-curricular)) OR (aerobic* or aikido or archery or athletics or badminton or ballgame* or "ball game*" or baseball or basketball or biathl* or bicycle* or bicycling or bike or biking or bowling or boxing or calisthenic* or "cardiopulmonary conditioning" or callisthenic* or canoe* or cricket or curling or cycling or danc* or diving or exercise* or fencing or floorball or "floor ball" or football or golf* or jogging or jumping or handball or hopping or gigong or "gi gong" or gymnastic* or hiit or hockey or "horseback riding" or "horse riding" or "isometric climbing" or jogging or judo or jujitsu or karate or "kung fu" or kung or marathon* or "martial art*" or mountaineer* or "neuromuscular facilitation*" or "power lifting" or pilates or plyometric* or racquetball or racketball or "racket ball" or rowing or rugby or running or sail* or skateboard* or skating or skiing or snowboard* or soccer or softball or squash or sport* or stretching or plyometric* or swim* or "tae kwon do" or taekwondo or taiji or taijiquan or taichi or walk* or warm-up or yoga or "multidisciplinary rehabilitation" or sport* or relaxation* or volleyball or walking or "weight* lifting" or "lifting weight*" or weightlifting or "isometric climbing" or wrestling or sedentary or inactiv* or sitting or stationary or TV or television) OR ((screen or media or computer or video or internet or web or outdoor) NEAR/3 time) OR ((media or computer or video or internet or electronic or web or outdoor) NEAR/3 (game* or gaming or play*)) OR ((media or computer or video or internet or web or "smart phone" or smartphone) NEAR/1 "use"))))

AND

((MAINSUBJECT.EXACT("Observation") OR MAINSUBJECT.EXACT("Cohort Analysis") OR MAINSUBJECT.EXACT.EXPLODE("Longitudinal Studies") OR MAINSUBJECT.EXACT("Quasiexperimental Design") OR MAINSUBJECT.EXACT("Policy") OR MAINSUBJECT.EXACT.EXPLODE("Public Policy") OR MAINSUBJECT.EXACT("School Policy") OR MAINSUBJECT.EXACT("Politics") OR MAINSUBJECT.EXACT("Politics of Education") OR MAINSUBJECT.EXACT("Policy Formation")) OR (ti(trial or "Case control" or (cohort NEAR/1 (study or studies)) or "Cohort analy*" or (("Follow up" or Followup) NEAR/1 (study or studies)) or (observational NEAR/1 (study or studies)) or Longitudinal or Retrospective or Prospective or "Cross sectional" or Quasi-Experimental or policy or policies or politic* or "action plan*" or regulat*) OR ab(random* or placebo or "Case control" or (cohort NEAR/1 (study or studies)) or (("Follow up" or Followup) NEAR/1 (study or studies)) or (observational NEAR/1 (study or studies)) or Longitudinal or Retrospective or Prospective or "Cross sectional" or Quasi-Experimental or policy or policies or politic* or "action plan*" or regulat*)))

Applied filters:

2009-01-01 - 2019-12-31

English (No articles in Swedish)

5. Sociological Abstracts (ProQuest)

Date of Search: 24 October 2019<br><br>Number of hits: 397 (original search 295)<br><br>Comments:	Field labels:<br>• MAINSUBJECT.EXACT.EXPLODE = exploded controlled term<br>• MAINSUBJECT.EXACT = non exploded controlled term<br>• ti = title<br>• ab = abstract<br>• NEAR/x = within x words, regardless of order<br>• * = truncation of word for alternate endings

((MAINSUBJECT.EXACT("Schools") OR MAINSUBJECT.EXACT("Elementary Schools") OR MAINSUBJECT.EXACT("Private Schools") OR MAINSUBJECT.EXACT("Public Schools") OR MAINSUBJECT.EXACT.EXPLODE("Secondary Schools") OR MAINSUBJECT.EXACT("School Environment")) OR (ti(school* or highschool* or grade-6 or grade-six or grade-7 or grade-seven or grade-8 or grade-eight or grade-9 or grade-nine or grade-10 or grade-11 or grade-12 or student* or pupil*) OR ab(school* or highschool* or grade-6 or grade-six or grade-7 or grade-seven or grade-8 or grade-eight or grade-9 or grade-nine or grade-10 or grade-11 or grade-12 or student* or pupil*)))

AND

((MAINSUBJECT.EXACT("Mental Illness") OR MAINSUBJECT.EXACT.EXPLODE("Affective Illness") OR MAINSUBJECT.EXACT("Mental Health") OR MAINSUBJECT.EXACT("Psychopathology") OR MAINSUBJECT.EXACT("Adjustment") OR MAINSUBJECT.EXACT("Anxiety") OR MAINSUBJECT.EXACT("Fear") OR MAINSUBJECT.EXACT("Phobias") OR MAINSUBJECT.EXACT("Compulsivity") OR MAINSUBJECT.EXACT("Well Being") OR MAINSUBJECT.EXACT("Quality of Life") OR MAINSUBJECT.EXACT("Satisfaction") OR MAINSUBJECT.EXACT("Life Satisfaction") OR MAINSUBJECT.EXACT("Happiness") OR MAINSUBJECT.EXACT("Pessimism") OR MAINSUBJECT.EXACT.EXPLODE("Self Concept") OR MAINSUBJECT.EXACT("Coping") OR MAINSUBJECT.EXACT("Resilience")) OR (ti(((mental or emotional) NEAR/3 (health or disorder* or disease* or problem* or symptom* or illness* or "ill health" or illhealth or distress or instabilit*)) OR ((psychiatric or psychologic) NEAR/3 (problem* or symptom* or disorder* or "ill health" or illhealth or distress or illness*)) OR (psychopathology or psychosocial or "abnormal psychology" or "internal distress" or internalising or internalizing or "adjustment disorder*" or "affective symptom*" or "affective problem*" or "affective distress" or mood or moods or "affective disorde*" or "affective syndrome*" or depress* or dysthymi* or sad or sadness or unhapp* or hopeless* or loneliness or lonely or worries or worry or "social withdrawal" or phobia or phobic or claustrophobia or agoraphob* or ophidiophobia or "school phobia" or neophobia or "avoidant disorder*" or overanxious or nervousness or "panic disorder*" or "panic attack*" or compulsive or compulsion or compulsiveness or obsess* or anankastic or "intrusive thoughts" or "intrusive thinking" or fear or avoidance or pessimism or pessimistic or despondency or despair

or frustrat* or anxiety or anxious) OR (well-being or wellbeing or wellness or optimis* or cheerful* or contentment or elated or elation or joy or enjoyment or "good feeling*" or "good mood" or happiness or happy or satisfaction or "quality of life" or HRQoL or QoL or "sense of coherence" or resilience or coping) OR (positive NEAR/3 (affect* or emotion* or mood*)) OR (self NEAR/3 (concept* or perception* or acceptance or confidence or esteem* or image or efficacy or reliance or worth or compassion))) OR ab(((mental or emotional) NEAR/3 (health or disorder* or disease* or problem* or symptom* or illness* or "ill health" or illhealth or distress or instabilit*)) OR ((psychiatric or psychologic) NEAR/3 (problem* or symptom* or disorder* or "ill health" or illhealth or distress or illness*)) OR (psychopathology or psychosocial or "abnormal psychology" or "internal distress" or internalising or internalizing or "adjustment disorder*" or "affective symptom*" or "affective problem*" or "affective distress" or mood or moods or "affective disorde*" or "affective syndrome*" or depress* or dysthymi* or sad or sadness or unhapp* or hopeless* or loneliness or lonely or worries or worry or "social withdrawal" or phobia or phobic or claustrophobia or agoraphob* or ophidiophobia or "school phobia" or neophobia or "avoidant disorder*" or overanxious or nervousness or "panic disorder*" or "panic attack*" or compulsive or compulsion or compulsiveness or obsess* or anankastic or "intrusive thoughts" or "intrusive thinking" or fear or avoidance or pessimism or pessimistic or despondency or despair or frustrat* or anxiety or anxious) OR (well-being or wellbeing or wellness or optimis* or cheerful* or contentment or elated or elation or joy or enjoyment or "good feeling*" or "good mood" or happiness or happy or satisfaction or "quality of life" or HRQoL or QoL or "sense of coherence" or resilience or coping) OR (positive NEAR/3 (affect* or emotion* or mood*)) OR (self NEAR/3 (concept* or perception* or acceptance or confidence or esteem* or image or efficacy or reliance or worth or compassion)))))

AND

((MAINSUBJECT.EXACT("Physical Education") OR MAINSUBJECT.EXACT("Physical Fitness") OR MAINSUBJECT.EXACT("Sports") OR MAINSUBJECT.EXACT("Professional Sports") OR MAINSUBJECT.EXACT("Leisure") OR MAINSUBJECT.EXACT.EXPLODE("Recreation") OR MAINSUBJECT.EXACT("Play") OR MAINSUBJECT.EXACT("Games") OR MAINSUBJECT.EXACT("Television Viewing") OR MAINSUBJECT.EXACT("Television") OR MAINSUBJECT.EXACT("Computers") OR MAINSUBJECT.EXACT("Internet")) OR (ti((physical* NEAR/2 (fitness or education or condition or mobility or activ* or effort*)) OR (activ* NEAR/3 (commut* or pause* or break* or lesson* or recess* or transport* or travel* or play* or movement* or lifestyle or locomotor or leisure or extracurricular or extra-curricular)) OR (aerobic* or aikido or archery or athletics or badminton or ballgame* or "ball game*" or baseball or basketball or biathl* or bicycle* or bicycling or bike or biking or bowling or boxing or calisthenic* or "cardiopulmonary conditioning" or callisthenic* or canoe* or cricket or curling or cycling or danc* or diving or exercise* or fencing or floorball or "floor ball" or football or golf* or jogging or jumping or handball or hopping or gigong or "gi gong" or gymnastic* or hiit or hockey or "horseback riding" or "horse riding" or "isometric climbing" or jogging or judo or jujitsu or karate or "kung fu" or kung or marathon* or "martial art*" or mountaineer* or "neuromuscular facilitation*" or "power lifting" or pilates or plyometric* or racquetball or racketball or "racket ball" or rowing or rugby or running or sail* or skateboard* or skating or skiing or snowboard* or soccer or softball or squash or sport* or stretching or plyometric* or swim* or "tae kwon do" or taekwondo or taiji or taijiquan or taichi or walk* or warm-up or yoga or "multidisciplinary rehabilitation" or sport* or relaxation* or volleyball or walking or "weight* lifting" or "lifting weight*" or weightlifting or "isometric climbing" or wrestling or sedentary or inactiv* or sitting or stationary or TV or television) OR ((screen or media or computer or video or internet or web or outdoor) NEAR/3 time) OR ((media or computer or video or internet or electronic or web or outdoor) NEAR/3 (game* or gaming or

play*)) OR ((media or computer or video or internet or web or "smart phone" or smartphone) NEAR/1 "use")) OR ab((physical* NEAR/2 (fitness or education or condition or mobility or activ* or effort*)) OR (activ* NEAR/3 (commut* or pause* or break* or lesson* or recess* or transport* or travel* or play* or movement* or lifestyle or locomotor or leisure or extracurricular or extra-curricular)) OR (aerobic* or aikido or archery or athletics or badminton or ballgame* or "ball game*" or baseball or basketball or biathl* or bicycle* or bicycling or bike or biking or bowling or boxing or calisthenic* or "cardiopulmonary conditioning" or callisthenic* or canoe* or cricket or curling or cycling or danc* or diving or exercise* or fencing or floorball or "floor ball" or football or golf* or jogging or jumping or handball or hopping or gigong or "gi gong" or gymnastic* or hiit or hockey or "horseback riding" or "horse riding" or "isometric climbing" or jogging or judo or jujitsu or karate or "kung fu" or kung or marathon* or "martial art*" or mountaineer* or "neuromuscular facilitation*" or "power lifting" or pilates or plyometric* or racquetball or racketball or "racket ball" or rowing or rugby or running or sail* or skateboard* or skating or skiing or snowboard* or soccer or softball or squash or sport* or stretching or plyometric* or swim* or "tae kwon do" or taekwondo or taiji or taijiquan or taichi or walk* or warm-up or yoga or "multidisciplinary rehabilitation" or sport* or relaxation* or volleyball or walking or "weight* lifting" or "lifting weight*" or weightlifting or "isometric climbing" or wrestling or sedentary or inactiv* or sitting or stationary or TV or television) OR ((screen or media or computer or video or internet or web or outdoor) NEAR/3 time) OR ((media or computer or video or internet or electronic or web or outdoor) NEAR/3 (game* or gaming or play*)) OR ((media or computer or video or internet or web or "smart phone" or smartphone) NEAR/1 "use"))))

AND

((MAINSUBJECT.EXACT.EXPLODE("Observation") OR MAINSUBJECT.EXACT("Cohort Analysis") OR MAINSUBJECT.EXACT("Longitudinal Studies") OR MAINSUBJECT.EXACT("Health Policy") OR MAINSUBJECT.EXACT("Policy") OR MAINSUBJECT.EXACT("Public Policy") OR MAINSUBJECT.EXACT("Government Policy") OR MAINSUBJECT.EXACT("Social Policy") OR MAINSUBJECT.EXACT("Politics") OR MAINSUBJECT.EXACT("Policy Making")) OR (ti(trial or "Case control" or (cohort NEAR/1 (study or studies)) or "Cohort analy*" or (("Follow up" or Followup) NEAR/1 (study or studies)) or (observational NEAR/1 (study or studies)) or Longitudinal or Retrospective or Prospective or "Cross sectional" or Quasi-Experimental or policy or policies or politic* or "action plan*" or regulat*) OR ab(random* or placebo or "Case control" or (cohort NEAR/1 (study or studies)) or (("Follow up" or Followup) NEAR/1 (study or studies)) or (observational NEAR/1 (study or studies)) or Longitudinal or Retrospective or Prospective or "Cross sectional" or Quasi-Experimental or policy or policies or politic* or "action plan*" or regulat*)))

Applied filters:

2009-01-01 - 2019-12-31

English (No articles in Swedish)
