# Supplementary material for: School-related physical activity interventions and mental health among children: a systematic review and meta-analysis
Source: Sports Med Open. 2020 Jun 16;6:25. doi: 10.1186/s40798-020-00254-x (PMC7297899; doi:10.1186/s40798-020-00254-x)
Supplement: Supplementary file 2 — Additional file 2. Online resource 2. Excluded studies. [file 40798_2020_254_MOESM2_ESM.docx]

| **Online resource 2. Excluded studies** | |
| --- | --- |
| **Publication** | **Reason for exclusion** |
| Ageret al. [1] | Not relevant intervention |
| Aittasalo et al. [2] | Not relevant outcome |
| Annesi et al. [3] | Not in school environment |
| Annesi et al. [4] | Not in school environment |
| Babic et al. [5] | Clinical population, inactive children |
| Barber et al. [6] | Preschool children |
| Barton et al. [7] | No control group |
| Bunketorp et al. [8] | No pre-intervention measurement |
| Busch et al. [9] | Not relevant intervention |
| Carlin et al. [10] | Not relevant outcome |
| Contento et al. [11] | Not relevant intervention |
| Daly et al. [12] | Not relevant outcome (ERICA) |
| De Bock et al. [13] | Preschool children |
| Donnelly et al. [14] | Not relevant outcome |
| Duncan et al. [15] | Not relevant outcome |
| Dunker et al. [16] | Clinical population |
| Dzewaltowski et al. [17] | Not relevant intervention |
| Ehud et al. [18] | Clinical population |
| Schäfer Elinder et al. [19] | Not relevant intervention |
| Fairclough et al. [20] | Not relevant outcome |
| Franco and Coteron [21] | Not relevant outcome |
| Frank et al. [22] | Clinical population |
| Gonzalez- Cutre et al. [23] | Not relevant outcome |
| Grillich et al. [24] | Not relevant intervention |
| Hartmann et al. [25] | No relevant results reported |
| Habib-Mourad et al. [26] | Not relevant outcome |
| Heidary et al. [27] | Conference paper |
| Hopkins et al. [28] | Not relevant outcome |
| Hsieh and Chen [29] | Not school-related |
| Hoying [30] | No control group |
| Huang et al. [31] | Not relevant outcome |
| Kennedy et al. [32] | Not relevant outcome |
| Kruger and Seng [33] | Not relevant outcome |
| Kriemler et al. [34] | No relevant results reported |
| Laberge et al. [35] | Clinical population |
| Lance [36] | Dissertation abstract |
| Lindqvist et al. [37] | Not relevant outcome |
| Lubans et al. [38] | Clinical population, inactive boys |
| Malakellis et al. [39] | No relevant results reported |
| Mantilla [40] | Not relevant outcome |
| Meyer et al. [41] | Not in school environment |
| Nagy et al. [42] | Not in school environment |
| Nathan et al. [43] | No pre-intervention measurement |
| Neumark-Sztainer et al. [44] | Clinical population |
| Reed et al. [45] | No control group |
| Rosenkranz et al. [46] | Study protocol |
| Rostami-Moez et al. [47] | Not relevant outcome |
| Saadat et al. [48] | No relevant outcome reported |
| Santos et al. [49] | Not relevant outcome |
| Sarkissian et al. [50] | No control group |
| Smith and Holloman [51] | Not relevant outcome |
| Telles et al. [52] | No control group, yoga vs physical activity |
| Tennant et al. [53] | Not in school environment |
| Thompson et al. [54] | Not relevant outcome |
| Tymms et al. [55] | Not relevant intervention |
| van Stralen et al. [56] | Not relevant outcome |
| Velasques et al. [57] | No relevant results reported |
| Watanabe et al. [58] | Not relevant outcome |
| Wong et al. [59] | Not in school environment |

1. Ager, A., et al., *The impact of the school-based Psychosocial Structured Activities (PSSA) program on conflict-affected children in Northern Uganda.* Journal of Child Psychology & Psychiatry & Allied Disciplines, 2011. **52**(11): p. 1124-33.

2. Aittasalo, M., et al., *Kids Out; evaluation of a brief multimodal cluster randomized intervention integrated in health education lessons to increase physical activity and reduce sedentary behavior among eighth graders.* BMC Public Health, 2019. **19**(1): p. 415.

3. Annesi, J.J., S.M. Walsh, and B.L. Greenwood, *Increasing Children's Voluntary Physical Activity Outside of School Hours Through Targeting Social Cognitive Theory Variables.* Journal of Primary Care & Community Health, 2016. **7**(4): p. 234-41.

4. Annesi, J.J., et al., *Effects of the Youth Fit 4 Life physical activity/nutrition protocol on body mass index, fitness and targeted social cognitive theory variables in 9- to 12-year-olds during after-school care.* Journal of Paediatrics & Child Health, 2017. **53**(4): p. 365-373.

5. Babic, M.J., et al., *Intervention to reduce recreational screen-time in adolescents: Outcomes and mediators from the 'Switch-Off 4 Healthy Minds' (S4HM) cluster randomized controlled trial.* Preventive Medicine, 2016. **91**: p. 50-57.

6. Barber, S.E., et al., *Assessing the feasibility of evaluating and delivering a physical activity intervention for pre-school children: a pilot randomised controlled trial.* Pilot & Feasibility Studies, 2016. **2**: p. 12.

7. Barton, J., et al., *The effect of playground- and nature-based playtime interventions on physical activity and self-esteem in UK school children.* Int J Environ Health Res, 2015. **25**(2): p. 196-206.

8. Bunketorp Kall, L., et al., *Effects of a Curricular Physical Activity Intervention on Children's School Performance, Wellness, and Brain Development.* Journal of School Health, 2015. **85**(10): p. 704-13.

9. Busch, V., R.J. De Leeuw, and A.J. Schrijvers, *Results of a multibehavioral health-promoting school pilot intervention in a Dutch secondary school.* Journal of Adolescent Health, 2013. **52**(4): p. 400-6.

10. Carlin, A., et al., *Effects of a peer-led Walking In ScHools intervention (the WISH study) on physical activity levels of adolescent girls: a cluster randomised pilot study.* Trials [Electronic Resource], 2018. **19**(1): p. 31.

11. Contento, I.R., et al., *Adolescents demonstrate improvement in obesity risk behaviors after completion of choice, control & change, a curriculum addressing personal agency and autonomous motivation.* Journal of the American Dietetic Association, 2010. **110**(12): p. 1830-9.

12. Daly, L.A., et al., *Yoga and Emotion Regulation in High School Students: A Randomized Controlled Trial.* Evidence-Based Complementary & Alternative Medicine: eCAM, 2015. **2015**: p. 794928.

13. De Bock, F., et al., *A participatory physical activity intervention in preschools: a cluster randomized controlled trial.* American Journal of Preventive Medicine, 2013. **45**(1): p. 64-74.

14. Donnelly, J.E., et al., *Physical activity and academic achievement across the curriculum: Results from a 3-year cluster-randomized trial.* Preventive Medicine, 2017. **99**: p. 140-145.

15. Duncan, M.J., Y. Al-Nakeeb, and A.M. Nevill, *Effects of a 6-week circuit training intervention on body esteem and body mass index in British primary school children.* Body Image, 2009. **6**(3): p. 216-20.

16. Dunker, K.L.L. and A.M. Claudino, *Preventing weight-related problems among adolescent girls: A cluster randomized trial comparing the Brazilian 'New Moves' program versus observation.* Obesity Research & Clinical Practice, 2018. **12**(1): p. 102-115.

17. Dzewaltowski, D.A., et al., *Healthy youth places: a randomized controlled trial to determine the effectiveness of facilitating adult and youth leaders to promote physical activity and fruit and vegetable consumption in middle schools.* Health Education & Behavior, 2009. **36**(3): p. 583-600.

18. Ehud, M., B.D. An, and S. Avshalom, *Here and now: Yoga in Israeli schools.* Int J Yoga, 2010. **3**(2): p. 42-7.

19. Elinder, L.S., et al., *A participatory and capacity-building approach to healthy eating and physical activity- SCIP-school: a 2-year controlled trial.* International Journal of Behavioral Nutrition & Physical Activity, 2012. **9**: p. 145.

20. Fairclough, S.J., et al., *A non-equivalent group pilot trial of a school-based physical activity and fitness intervention for 10-11 year old english children: born to move.* BMC Public Health, 2016. **16**(1): p. 861.

21. Franco, E. and J. Coteron, *The Effects of a Physical Education Intervention to Support the Satisfaction of Basic Psychological Needs on the Motivation and Intentions to be Physically Active.* Journal of Human Kinetics, 2017. **59**: p. 5-15.

22. Frank, J.L., B. Bose, and A. Schrobenhauser-Clonan, *Effectiveness of a School-Based Yoga Program on Adolescent Mental Health, Stress Coping Strategies, and Attitudes toward Violence: Findings from a High-Risk Sample.* Journal of Applied School Psychology, 2014. **30**(1): p. 29-49.

23. Gonzalez-Cutre, D., et al., *A school-based motivational intervention to promote physical activity from a self-determination theory perspective.* The Journal of Educational Research, 2018. **111**(3): p. 320-330.

24. Grillich, L., et al., *Effectiveness evaluation of a health promotion programme in primary schools: a cluster randomised controlled trial.* BMC Public Health, 2016. **16**: p. 679.

25. Hartmann, T., et al., *Effects of a school-based physical activity program on physical and psychosocial quality of life in elementary school children: a cluster-randomized trial.* Pediatric Exercise Science, 2010. **22**(4): p. 511-22.

26. Habib-Mourad, C., et al., *Promoting healthy eating and physical activity among school children: findings from Health-E-PALS, the first pilot intervention from Lebanon.* BMC Public Health, 2014. **14**: p. 940.

27. Heidary, A., et al., *Effects of aerobic exercise on anxiety*, in *2nd World Conference on Psychology, Counselling and Guidance-2011*, D.E. Ongen, et al., Editors. 2011.

28. Hopkins, L.C., et al., *Participation in structured programming may prevent unhealthy weight gain during the summer in school-aged children from low-income neighbourhoods: feasibility, fidelity and preliminary efficacy findings from the Camp NERF study.* Public Health Nutrition, 2019. **22**(6): p. 1100-1112.

29. Hsieh, C.Y. and T. Chen, *Effect of Pokemon GO on the Cognitive Performance and Emotional Intelligence of Primary School Students.* Journal of Educational Computing Research, 2019. **57**(7): p. 1849-1874.

30. Hoying, J.A., *COPE: A pilot study with urban sixth grade youth to improve physical activity and mental health outcomes.* Dissertation Abstracts International: Section B: The Sciences and Engineering, 2017. **77**(10-B(E)): p. No Pagination Specified.

31. Huang, C., et al., *Changes in Self-Efficacy and Outcome Expectations From Child Participation in Bicycle Trains for Commuting to and From School.* Health Education & Behavior, 2018. **45**(5): p. 748-755.

32. Kennedy, S.G., et al., *Implementing Resistance Training in Secondary Schools: A Cluster Randomized Controlled Trial.* Medicine & Science in Sports & Exercise, 2018. **50**(1): p. 62-72.

33. Kruger, M. and C. Seng, *Effects of Short Practice of Climbing on Barriers Self-Efficacy within a Physical Education and Sport Intervention in Germany.* Sports, 2019. **7**(4): p. 04.

34. Kriemler, S., et al., *Effect of school based physical activity programme (KISS) on fitness and adiposity in primary schoolchildren: cluster randomised controlled trial.* BMJ, 2010. **340**.

35. Laberge, S., P.L. Bush, and M. Chagnon, *Effects of a culturally tailored physical activity promotion program on selected self-regulation skills and attitudes in adolescents of an underserved, multiethnic milieu.* American Journal of Health Promotion, 2012. **26**(4): p. e105-15.

36. Lance, M.M., *Yoga in schools: A tool for reducing anxiety.* Dissertation Abstracts International Section A: Humanities and Social Sciences, 2012. **72**(12-A): p. 4471.

37. Lindqvist, A.K., et al., *Moving from idea to action: promoting physical activity by empowering adolescents.* Health Promotion Practice, 2014. **15**(6): p. 812-8.

38. Lubans, D.R., et al., *Mediators of Psychological Well-being in Adolescent Boys.* Journal of Adolescent Health, 2016. **58**(2): p. 230-6.

39. Malakellis, M., et al., *School-based systems change for obesity prevention in adolescents: outcomes of the Australian Capital Territory 'It's Your Move!'.* Australian & New Zealand Journal of Public Health, 2017. **41**(5): p. 490-496.

40. Mantilla, C., *Comparison between a comprehensive wellness-based afterschool program and a traditional ymca after-school program on measures of physical fitness, health-related, and executive cognitive function variables in minority elementary school children.* Dissertation Abstracts International: Section B: The Sciences and Engineering, 2016. **76**(7-B(E)): p. No Pagination Specified.

41. Meyer, U., et al., *Long-term effect of a school-based physical activity program (KISS) on fitness and adiposity in children: a cluster-randomized controlled trial.* PLoS ONE [Electronic Resource], 2014. **9**(2).

42. Nagy, M.R., et al., *Affective Responses to Intermittent Physical Activity in Healthy Weight and Overweight/Obese Elementary School-Age Children.* Journal of Physical Activity & Health, 2017. **14**(11): p. 845-851.

43. Nathan, S., et al., *"We wouldn't of made friends if we didn't come to Football United": the impacts of a football program on young people's peer, prosocial and cross-cultural relationships.* BMC Public Health, 2013. **13**: p. 399.

44. Neumark-Sztainer, D.R., et al., *New moves-preventing weight-related problems in adolescent girls a group-randomized study.* American Journal of Preventive Medicine, 2010. **39**(5): p. 421-32.

45. Reed, K., et al., *A repeated measures experiment of green exercise to improve self-esteem in UK school children.* PLoS ONE [Electronic Resource], 2013. **8**(7): p. e69176.

46. Rosenkranz, R.R., et al., *A cluster-randomized controlled trial of strategies to increase adolescents' physical activity and motivation during physical education lessons: the Motivating Active Learning in Physical Education (MALP) trial.* BMC Public Health, 2012. **12**: p. 834.

47. Rostami-Moez, M., et al., *Effect of Educational Program, Based on PRECEDE and Trans-Theoretical Models, on Preventing Decline in Regular Physical Activity and Improving it among Students.* Journal of Research in Health Sciences, 2017. **17**(2): p. e00375.

48. Saadat, M., A.H. Motlagh, and A. Alamdari, *Investigating the Role of Aerobic Exercise on Subjective Well-Being among High School Girls in Yasuj High School in 2017.* World Family Medicine, 2018. **16**(1): p. 250-253.

49. Santos, R.G., et al., *Effectiveness of peer-based healthy living lesson plans on anthropometric measures and physical activity in elementary school students: a cluster randomized trial.[Erratum appears in JAMA Pediatr. 2015 Jan;169(1):96].* JAMA Pediatrics, 2014. **168**(4): p. 330-7.

50. Sarkissian, M., et al., *Effects of a Kundalini Yoga Program on Elementary and Middle School Students' Stress, Affect, and Resilience.* Journal of Developmental and Behavioral Pediatrics, 2018. **39**(3): p. 210-216.

51. Smith, L.H. and C. Holloman, *Comparing the effects of teen mentors to adult teachers on child lifestyle behaviors and health outcomes in Appalachia.* Journal of School Nursing, 2013. **29**(5): p. 386-96.

52. Telles, S., et al., *Effect of yoga or physical exercise on physical, cognitive and emotional measures in children: a randomized controlled trial.* Child & Adolescent Psychiatry & Mental Health [Electronic Resource], 2013. **7**(1): p. 37.

53. Tennant, R.G., et al., *Preventing Internalizing Problems in Young Children: A Randomized Controlled Trial of the Feelings and Friends (Year 3) Program with a Motor Skills Component.* Frontiers in Psychology, 2017. **8**: p. 291.

54. Thompson, H.R., et al., *The impact of moderate-vigorous intensity physical education class immediately prior to standardized testing on student test-taking behaviors.* Mental Health and Physical Activity, 2016. **11**: p. 7-12.

55. Tymms, P.B., et al., *Clustered randomised controlled trial of two education interventions designed to increase physical activity and well-being of secondary school students: the MOVE Project.* BMJ Open, 2016. **6**(1): p. e009318.

56. van Stralen, M.M., et al., *Mediators of the effect of the JUMP-in intervention on physical activity and sedentary behavior in Dutch primary schoolchildren from disadvantaged neighborhoods.* International Journal of Behavioral Nutrition & Physical Activity, 2012. **9**: p. 131.

57. Velasquez, A.M., et al., *Yoga for the prevention of depression, anxiety, and aggression and the promotion of socio-emotional competencies in school-aged children.* Educational Research and Evaluation, 2015. **21**(5-6): p. 407-421.

58. Watanabe, J., et al., *Effect of School-Based Home-Collaborative Lifestyle Education on Reducing Subjective Psychosomatic Symptoms in Adolescents: A Cluster Randomised Controlled Trial.* Plos One, 2016. **11**(10).

59. Wong, W.W., et al., *A Community-based Healthy Living Promotion Program Improved Self-esteem Among Minority Children.* Journal of Pediatric Gastroenterology & Nutrition, 2016. **63**(1): p. 106-12.
