# Supplementary material for: School-related physical activity interventions and mental health among children: a systematic review and meta-analysis
Source: Sports Med Open. 2020 Jun 16;6:25. doi: 10.1186/s40798-020-00254-x (PMC7297899; doi:10.1186/s40798-020-00254-x)
Supplement: Supplementary file 3 — Additional file 3. Online resource 3. Implementation factors. [file 40798_2020_254_MOESM3_ESM.docx]

| **Online resource 3. Implementation factors**   \|  \|  \|  \|  \|  \|  \|  \|  \| \| --- \| --- \| --- \| --- \| --- \| --- \| --- \| --- \| \| **Author** \| **Fidelity** \| **Measurement of dose** \| **Dose delivered or received** \| **Reach^a^** \| **Responsiveness** \| **Adapta-tion** \| **Comment** \| \| Adab et al. [24] \| "Schools were classified into three groups (low, medium and high) based on the level of overall implementation". (Low fidelity for PA component) \| Observation and interviews \| Only 17% of schools included in process evaluation obtained high implementation and high fidelity score regarding PA component. Variation in delivery and perceived impact of intervention components \| 3=low \| The intervention was well received and valued at various levels by schools, children and their parents. Teachers experienced difficulties to fit it in \| NR \|  \| \| Altunkurek and Bebis [25] \| NR* \| NR \| NR \| 1=high \| NR \| NR \|  \| \| Ardic and Erdogan [26] \| NR \| NR \| NR \| 4=unknown \| NR \| NR \|  \| \| Azevedo et al. [27] \| Dance mats provided to each intervention school and 6-weeks introduction programme \| Interviews with teachers and focus groups with pupils \| Substantial variation between schools. After 6 week introduction use became increasingly patchy within and across schools. Dance mats not used routinely and did not lead to increase in PA \| 3=low \| Pupils became bored after a while \| NR \| Data from Watson 2016 \| \| Bremer et al. [28] \| Number of weekly minutes in the programme, student engagement \| Teacher questionnaire post intervention regarding programme adherence and student behaviour \| Only 21% of teachers adhered fully to the programme (>100 min/week). Others reported 30-100 min/week), 3-4 days/week \| 3=low \| NR \| NR \|  \| \| Breslin et al. [29] \| NR \| NR \| NR \| 4=unknown \| NR \| NR \|  \| \| Casey et al. [30] \| High level of implementation but some aspects were not implemented as intended \| Teachers feedback on workshops, sessions performed and lesson implementation. Focus groups with teachers, student survey, field notes \| Teachers reported that lesson were implemented in line with the lesson plan ‘almost all’ (54.3%) or ‘most’ (24.4%) of the time. 91 completers; 271 non-completers \| 3=low \| Most students were positive, year 9 students were less positive. Implementation was sometimes negatively influenced by a lack of individual and organisational readiness to adopt program principles. \| Some restructuring of PE lessons occurred \| Data from Casey 2014 \| \| Christiansen et al. [31] \| Fidelity varied between schools, problems with “quality of delivery and compliance with pedagogical principles”  High adoption of PE and brain breaks, but low for recess activities \| Teacher questionnaire about adherence to program components, readiness, observations, interviews and written statements \| More than nine out of ten educators integrated brain breaks in their lessons and practically all the physical education teachers used the physical education lesson plans. The educators delivered on average 4.5 brain breaks per week and up to 90% of the physical education teachers used the project lesson plans for at least half of their classes. \| 1=high  no schools withdrew \| NR \| NR \| Data from Smedegaard 2017 \| \| Corder et al. [32] \| Fidelity not mentioned. The intervention was delivered to the whole of year 9 in both intervention schools by trained teachers with limited researcher assistance \| Questionnaire and focus groups with students but no formal analysis was done \| The intervention was delivered to the whole of year 9 in both intervention schools. It was a challenge for mentors to deliver the bulk of the intervention \| 2=medium \| Teachers enjoyed the programme, but a lot of work. Most student found it fun and said it would encouraged them to do more activity \| NR \|  \| \| Costigan et al. [33] \| Fidelity not mentioned but probably high. In regard to feasibility, the program achieved high recruitment, good adherence and retention \| % attendance \| On average 2.2 of 3 sessions per week attended \| 1=high \| 91% retention rate. Students reported that the programme was enjoyable \| No \| Data from Costigan 2015 \| \| Frank et al. [34] \| TheTransformative Life Skills curriculum provides sequenced scripts to support high-fidelity. On average 92 % of intervention components were implemented with fidelity \| Instructors completed checklist \| On average 92 % of intervention components were implemented with fidelity \| 1=high \| Above average acceptability \| No \|  \| \| Ha et al. [35] \| NR \| NR \| NR \| 4=unknown \| NR \| NR \|  \| \| Haden et al. [36] \| Fidelity not mentioned but probably high. Programme was delivered by certified yoga teachers. All classes integrated the eight limbs of yoga but in an indirect and varied manner \| A brief measure was created to assess the students’ level of engagement \| Reports that all participants received allocated intervention \| 1=high \| NR \| No \|  \| \| Halliwell et al.[37] \| All schools delivered one yoga session per week for 4 weeks by certified instructor \| Number of sessions delivered \| Same dose delivered to all schools according to program \| 1=high \| Yoga lessons were appreciated by the children \| No \|  \| \| Harrington et al. [38] \| Only 36% of girls had actually participated \| Feedback forms and attendance records were completed at the end of all teacher and peer leader training and review days.  Semi-structured interviews and focus groups with the lead girls \| Many schools did not achieve many of the activities on their action plans \| 3=low \| Exit survey by children, 46% of girls reported liking physical activity a bit or a lot more \| NR \| Gorely 2019 \| \| Hyndman et al. [40] \| School yard intervention implemented as intended \| Field notes and video recordings \| Children in intervention school were more physically active and less sedentary than in control school \| 1=high \| Children enjoyed the intervention \| No \|  \| \| Höner and Demetriou [39] \| Fidelity not mentioned but probably high. Detailed scripts were provided and necessary materials for the implementation of the lessons were given to the teachers. Teachers were instructed to teach all eight health-promotion PE lessons \| Observations \| Teachers implemented the lessons to a satisfactory extent \| 1=high \| Teachers were satisfied with the content of the lessons and the material. Students were from neutral to positive \| Minor \|  \| \| Khalsa et al. [41] \| Fidelity not mentioned but probably high. Standardised yoga program given by trained instructors \| % attendance, field notes by yoga instructors \| 70-80% attendance \| 2=medium \| Received positively by many students \| No \|  \| \| Lubans et al. [42] \| Intervention delivery fidelity was found to be 74.0% \| % attendance, observations \| >80 % received intervention \| 1=high \| Participants enjoyed the programme \| No \|  \| \| Luna et al. [43] \| The intervention “sports education model” followed a detailed programme and was led by teachers \| Weekly “blind” visits to schools by experts to verify correct implementation \| NR \| 1=high  Delivered in class \| NR \| No \|  \| \| Melnyk et al. [45] \| Fidelity not mentioned but probably high. The COPE TEEN program was delivered 2 to 3 days a week during the teen’s 9-week health class \| % attendance \| 88-92% attendance in sessions \| 1=high \| Students found the programme informative and helpful \| No \|  \| \| Melnyk et al. [44] and Melnyk et al. [46] \| Attention-control teachers (control) implemented their intervention with greater fidelity than COPE TEEN (intervention) teachers \| Observation, fidelity instrument developed for the study. Teachers recorded the tasks accomplished in each intervention session as well as time spent on each task, impressions of the flow, and content and acceptance of the sessions in an intervention diary. \| Manualised programme, implementation varied from <50% to >90% in sessions. Decreases in fidelity at least once in approximately half of the classrooms \| 1=high \| In general well received by students and parents. Teachers suggested some changes to the programme \| No \| Data from Kelly 2015 \| \| Moore et al. [47] \| NR \| NR \| 10 sessions for 10 weeks with 7 components delivered face-to-face by registered psychologist and taekwondo instructor \| 1= high  17% dropped out \| 75% of participants reported that they would like to continue with the activities \| No \|  \| \| Noggle et al. [48] \| Fidelity not mentioned but probably high. Delivered by trained yoga instructors \| % attendance and questionnaire \| 58% attendance \| 3=low \| "Fairly high" on student evaluation questionnaire \|  \|  \| \| Olive et al. [49] \| Programme delivered by specialist PE teachers. Fidelity not mentioned but probably high \| Observations regarding time spent active during lessons  Number of lessons per school year taught by specialist PE-teacher \| Time in moderate-to-vigorous physical activity (17 min/lesson), time for fitness activities (7.5 min), game play (11.2 min), general knowledge (8.4 min) all higher than control \| NR but young children in class, therefore probably 1=high \| Lack of variation of programme may have contributed to the program feeling “tired” \| No \|  \| \| Resaland et al. [50] \| High fidelity according to reported time for PA by teachers \| PA measured objectively \| According to protocol extra 165 min/week of PA. But no difference to control according to objective measurement \| 1=high \| NR \| No \|  \| \| Ruiz-Ariza et al. [51] \| High fidelity in final sample \| Maintenance of heart rate >85% of maximum during 80% of session \| 16 minutes of C-HIIT within PE classes 2 times per week \| 1=high \| NR \| No \|  \| \| Shannon et al. [52] \| Delivered in class but “lack of fidelity data” \| NR \| NR \| 4=unknown \| NR \| NR \|  \| \| Velez et al. [53] \| Fidelity not mentioned but probably high. Guided programme with 4 students per instructor from research team \| % attendance \| 30 of 36 sessions attended \| 1=high \| NR \| No \|  \| \| Yook et al. [54] \| NR \| NR \| NR \| 4=unknown \| NR \| NR \|  \| \|  \|  \|  \|  \|  \|  \|  \|  \| |  |  |  |  |
| --- | --- | --- | --- | --- | --- | --- | --- | --- | --- | --- | --- | --- | --- | --- | --- | --- | --- | --- | --- | --- | --- | --- | --- | --- | --- | --- | --- | --- | --- | --- | --- | --- | --- | --- | --- | --- | --- | --- | --- | --- | --- | --- | --- | --- | --- | --- | --- | --- | --- | --- | --- | --- | --- | --- | --- | --- | --- | --- | --- | --- | --- | --- | --- | --- | --- | --- | --- | --- | --- | --- | --- | --- | --- | --- | --- | --- | --- | --- | --- | --- | --- | --- | --- | --- | --- | --- | --- | --- | --- | --- | --- | --- | --- | --- | --- | --- | --- | --- | --- | --- | --- | --- | --- | --- | --- | --- | --- | --- | --- | --- | --- | --- | --- | --- | --- | --- | --- | --- | --- | --- | --- | --- | --- | --- | --- | --- | --- | --- | --- | --- | --- | --- | --- | --- | --- | --- | --- | --- | --- | --- | --- | --- | --- | --- | --- | --- | --- | --- | --- | --- | --- | --- | --- | --- | --- | --- | --- | --- | --- | --- | --- | --- | --- | --- | --- | --- | --- | --- | --- | --- | --- | --- | --- | --- | --- | --- | --- | --- | --- | --- | --- | --- | --- | --- | --- | --- | --- | --- | --- | --- | --- | --- | --- | --- | --- | --- | --- | --- | --- | --- | --- | --- | --- | --- | --- | --- | --- | --- | --- | --- | --- | --- | --- | --- | --- | --- | --- | --- | --- | --- | --- | --- | --- | --- | --- | --- | --- | --- | --- | --- | --- | --- | --- | --- | --- | --- | --- | --- | --- | --- | --- | --- | --- | --- | --- | --- | --- | --- | --- | --- | --- | --- | --- | --- | --- | --- | --- | --- | --- | --- | --- | --- | --- | --- | --- | --- | --- | --- |
