# Supplementary material for: School-related physical activity interventions and mental health among children: a systematic review and meta-analysis
Source: Sports Med Open. 2020 Jun 16;6:25. doi: 10.1186/s40798-020-00254-x (PMC7297899; doi:10.1186/s40798-020-00254-x)
Supplement: Supplementary file 4 — Additional file 4. Online resource 4. Outcomes and instruments. [file 40798_2020_254_MOESM4_ESM.docx]

| **Online resource 4. Outcomes and instruments** | | | |
| --- | --- | --- | --- |
|  | **Outcome** | **Instrument** | **Author** |
| 1 | Health-related quality of life | PedsQL - total score (PedsQL child 5-7 years self-report, PedsQL 4.0 generic core scale for teens, PedsQL 4.0 8-12 years) | Adab et al. [24], Casey et al. [30], Hyndmann et al. [40] |
|  |  | KINDL-R- total score | Höner and Demetriou [39] |
|  |  | KIDSCREEN-10 health-related quality of life | Ha et al. [35], Azevedo et al. [27],  Resaland et al. [50], Luna et al. [43] |
|  |  | KIDSCREEN-27 - health-related quality of life | Shannon et al. [52], Breslin et al. [29] |
|  |  | Child Health Utility- 9D | Harrington et al. [38] |
| 2 | Emotional problems | PedsQL- emotional functioning score | Adab et al. [24], Casey et al. [30] |
|  |  | KINDL-R- emotional | Höner and Demetriou [39] |
|  |  | SDQ- emotional problems | Bremer et al. [28], Moore et al. [47] |
| 3 | Anxiety | Beck youth inventory- anxiety | Ardic and Erdogan [26], Melnyk et al. 2009, 2013, 2015 [44-46] |
|  |  | BASC-2: anxiety | Khalsa et al. [41] |
|  |  | The Profile of Mood states-Short Form (Poms-SF)- tension axiety | Khalsa et al.* [41], Noggle et al. [48] |
|  |  | BASC-2- test anxiety | Khalsa et al. *[41] |
|  |  | Social Anxiety Scale for Adolescents | Luna et al. [43] |
| 4 | Depression | Beck youth inventory- depression | Ardic and Erdogan [26], Melnyk et al. 2009, 2013, 2015, [44-46] |
|  |  | The Profile of Mood states-Short Form (Poms-SF)- depression dejection | Khalsa et al. *[41], Noggle et al. [48] |
|  |  | BASC 2: depression | Khalsa et al. [41] |
|  |  | Children depression inventory | Olive et al. [49] |
| 5 | Well-being | Warwick-Edinburgh Wellbeing scale- total score | Corder et al. [32] |
|  |  | Flourishing scale- Total score | Costigan et al. [33] |
|  |  | Psychological well-being scale-happiness | Yook et al. [54] |
|  |  | KIDSCREEN-27 psychological wellbeing | Azevedo et al. [27], Ha et al. [35], Breslin et al. [29], Resaland et al. [50] |
|  |  |  |  |
|  |  | Subjective Happiness Scale | Bremer et al. [28] |
|  |  | Trait and Emotional Intelligence Questionaire short Form- well being | Ruiz-Ariza et al. [51] |
|  |  | The Five‐Factor Wellness Scale – Adolescent Form | Altunkurek and Bebis [25] |
| 6 | Self-esteem, self-worth | KINDL-R- self esteem | Höner and Demetriou [39] |
|  |  | The Self Perception Profile for Children (SPPC)- global self-worth | Haden et al. [36], Christiansen et al. [31] |
|  |  | Rosenberg Self-esteem Scale (RSE)- Total score | Yook et al. [54], Bremer et al. [28] |
|  |  | Marsh's physical self-description questionnaire | Lubans et al. [42] |
|  |  | BASC-2- self- esteem | Khalsa et al. [41] |
|  |  | Children and youth physical self-perception profile- global self-worth | Velez et al. [53] |
|  |  | Self-efficacy Questionaire for children- Total score | Moore et al. [47] |
|  |  | Physical self-perception profile questionnaire- Self-esteem | Harrington et al. [38] |
| 7 | Resilience | Resilience scale (RS)- total score | Khalsa et al. [41], Noogle et al. [48] |
|  |  | Ego-resiliency scale- total score | Yook et al. [54] |
|  |  | Child and Youth Recillience scale- total score | Moore et al. [47] |
| 8 | Positive affect | PANAS-C positive affect | Haden et al. [36], Frank et al. [34], Noggle et al. [48], Halliwell et al. [37], Luna et al. [43] |
| 9 | Negative affect | PANAS-C negative affect | Haden et al. [36], Frank et al. [34], Noggle et al. [48], Halliwell et al. [37], Luna et al. [43] |
| 10 | Internalising problems | Child Behavior Check List (CBCL)- internalising problems | Haden et al. [36] |
|  |  | Beck anxiety inventory | Ardic and Erdogan [26], Melnyk et al. 2009, 2013, 2015 [44-46] |
|  |  | BASC-2: anxiety | Khalsa et al. [41] |
|  |  | The Profile of Mood states-Short Form (Poms-SF)- tension Axiet | Khalsa et al.* [41], Noggle et al. [48] |
|  |  | Beck depression inventory- total score | Ardic and Erdogan [26], Melnyk, et al. 2009, 2013, 2015 [44-46] |
|  |  | The Profile of Mood states-Short Form (Poms-SF)- depression dejection | Khalsa et al.* [41], Noggle et al. [48] |
|  |  | BASC 2: depression | Khalsa et al. [41] |
|  |  | PedsQL- emotional functioning score | Adab et al. [24], Casey et al. [30] |
|  |  | KINDL-R- emotional | Höner and Demetriou [39] |
|  |  | Kessler Psychological Distress Scale (K10)- Total score | Costigan et al. [33] |
|  |  | PANAS-C negative affect | Haden et al.* [36], Frank et al. [34], Noggle et al.* [48], Halliwell et al. [37], Luna et al.* [43] |
|  |  | Basc2- test anxiety | Khalsa et al.* [41] |
|  |  | SDQ- emotional problems | Bremer et al. [28], Moore et al. [47] |
|  |  | Social Anxiety Scale for Adolescents | Luna et al. [43] |
|  |  | Children depression inventory | Olive et al. [49] |
| 11 | Positive mental health | PedsQL - total score (PedsQL child 5-7 years self report, PedsQL 4.0 generic core scale for teens, PedsQL 4.0 8-12 years) | Adab et al. [24], Casey et al. [30], Hyndmann et al. [40] |
|  |  | KINDL-R- total score | Höner and Demetriou [39] |
|  |  | KIDSCREEN-10 health-related quality of life | Ha et al. [35], Azevedo et al. [27] Luna et al. [43], Resaland et al. [50] |
|  |  | KIDSCREEN-27 total score | Breslin et al. [29], Shannon et al. [52] |
|  |  | Child Health Utility- 9D- Health related quality of life | Harrington et al. [38] |
|  |  | Warwick-Edinburgh Wellbeing scale- total score | Corder et al. [32] |
|  |  | Flourishing scale- Total score | Costigan et al. [33] |
|  |  | Psychological well-being scale-happiness | Yook et al.* [54] |
|  |  | KINDL-R- self esteem | Höner and Demetriou* [39] |
|  |  | The Self Perception Profile for Children (SPPC)- global self worth | Haden et al. [36] Christiansen et al. [31] |
|  |  | Rosenberg Self-esteem Scale (RSE)- Total score | Yook et al.* [54], Bremer et al. [28] |
|  |  | Marsh's physical self-description questionnaire | Lubans et al. [42] |
|  |  | BASC-2- self- esteem | Khalsa et al.* [41] |
|  |  | Resilience scale (RS)- total score | Khalsa et al. [41], Noogle et al. [48] |
|  |  | Ego-resiliency scale- total score | Yook et al. [54] |
|  |  | Child and Youth Recillience scale- total score | Moore et al. [47] |
|  |  | PANAS-C positive affect | Haden et al.* [36], Frank et al. [34], Noggle et al.* [48], Halliwell et al. [37], Luna et al.* [43] |
|  |  | Children and youth physical self-perception prolife- global self worth | Velez et al. [53] |
|  |  | The Five‐Factor Wellness Scale – Adolescent Form | Altunkurek and Bebis [25] |
|  |  | 4-item Subjective Happiness Scale | Bremer et al.* [28] |
|  |  | Physical self-perception profile questionnaire- Self-esteem | Harrington et al. [38] |
|  |  | Trait and Emotional Intelligence Questionaire short Form- well being | Ruiz- Ariza et al. [51] |
|  |  | *Not included in meta-analysis |  |
