# Supplementary material for: School-related physical activity interventions and mental health among children: a systematic review and meta-analysis
Source: Sports Med Open. 2020 Jun 16;6:25. doi: 10.1186/s40798-020-00254-x (PMC7297899; doi:10.1186/s40798-020-00254-x)
Supplement: Supplementary file 5 — Additional file 5. Online resource 5. Quality assessment. [file 40798_2020_254_MOESM5_ESM.docx]

| **Online resource 5. Quality assessment of included studies with the Effective Public Health Practice Project tool (EPHPP)** | | | | | | | | | |
| --- | --- | --- | --- | --- | --- | --- | --- | --- | --- |
| **Author, year** | **Selection bias** | **Study design** | **Confounders** | **Blinding** | **Data collection method** | **Withdrawals and dropouts** | **Intervention integrity measured (Q2)** | **Analyses Intention to treat** | **Global rating** |
| Adab et al. [24] | moderate | strong | strong | moderate | strong | weak | yes | yes | moderate |
| Altunkurek and Bebis [25] | weak | strong | strong | weak | strong | strong | no | NR | weak |
| Ardic and Erdogan [26] | moderate | strong | strong | weak | strong | moderate | no | NR | moderate |
| Azevedo et al. [27] | weak | strong | strong | weak | strong | moderate | yes | NR | weak |
| Bremer et al. [28] | weak | strong | strong | weak | strong | strong | yes | NR | weak |
| Breslin et al. [29] | moderate | strong | weak | weak | strong | strong | no | NR | weak |
| Casey et al. [30] | weak | strong | strong | moderate | strong | moderate | yes | partly ITT | moderate |
| Christiansen et al. [31] | strong | strong | weak | strong | strong | moderate | yes | yes | moderate |
| Corder [32] | moderate | strong | weak | moderate | strong | strong | no | NR | moderate |
| Costigan et al. [33] | moderate | strong | strong | moderate | strong | strong | yes | NR | strong |
| Frank et al. [34] | weak | strong | strong | moderate | strong | strong | yes | yes | moderate |
| Ha et al. [35] | moderate | strong | strong | moderate | strong | strong | no | NR | strong |
| Haden et al. [36] | weak | strong | strong | moderate | strong | strong | yes | NR | moderate |
| Halliwell et al.[37] | moderate | strong | strong | weak | strong | moderate | no | NR | moderate |
| Harrington et al. [38] | moderate | strong | strong | weak | strong | moderate | yes | yes | moderate |
| Hyndman et al. [40] | weak | strong | strong | weak | strong | weak | yes | NR | weak |
| Höner and Demetriou [39] | moderate | strong | strong | moderate | strong | weak | yes | NR | moderate |
| Khalsa et al. [41] | moderate | strong | strong | weak | strong | moderate | yes | yes | moderate |
| Lubans et al. [42] | moderate | strong | strong | weak | strong | strong | yes | yes | moderate |
| Luna et al. [43] | weak | strong | weak | weak | strong | strong | no | NR | weak |
| Melnyk [45] | weak | strong | strong | weak | strong | strong | yes | no | weak |
| Melnyk et al., [44] & Melnyk et al [46] | weak | strong | strong | moderate | strong | moderate | yes | yes | moderate |
| Moore et al. [47] | moderate | strong | strong | moderate | strong | strong | no | NR | strong |
| Noggle et al. [48] | weak | strong | strong | weak | strong | strong | yes | yes | weak |
| Olive et al. [49] | strong | strong | strong | moderate | strong | strong | yes | yes | moderate |
| Resaland et al. [50] | strong | strong | strong | moderate | strong | strong | yes | yes | strong |
| Ruiz-Ariza et al. [51] | weak | strong | strong | moderate | strong | strong | yes | NR | moderate |
| Shannon et al. [52] | moderate | strong | weak | weak | strong | strong | no | NR | weak |
| Velez et al. [53] | moderate | strong | weak | weak | strong | strong | yes | no | weak |
| Yook et al. [54] | weak | strong | weak | moderate | strong | strong | no | NR | weak |

NR=not reported
